# Chemoprofiling and medicinal potential of underutilized leaves of *Cyperus scariosus*

**DOI:** 10.1038/s41598-024-58041-7

**Published:** 2024-03-27

**Authors:** Yashika Gandhi, Vijay Kumar, Gagandeep Singh, Shyam Baboo Prasad, Sujeet K. Mishra, Hemant Soni, Hemant Rawat, Simranjeet Singh, Vaibhav Charde, Akhil Gupta, Daljeet Singh Dhanjal, Sudhanshu Kumar Jha, Smriti Tandon, Prateeksha Bhagwat, Jagdish C. Arya, Praveen C. Ramamurthy, Rabinarayan Acharya, Ch. Venkata Narasimhaji, Arjun Singh, Ravindra Singh, Narayanam Srikanth, Thomas J. Webster

**Affiliations:** 1 Central Ayurveda Research Institute, Jhansi, Uttar Pradesh 284003 India; 2 Interdisciplinary Centre for Water Research, Indian Institute of Sciences, Bangalore, 560012 India; 3 Lovely Professional University, Phagwara, Punjab 144411 India; 4 Central Council for Research in Ayurvedic Sciences, New Delhi, 110058 India; 5 School of Health Sciences and Biomedical Engineering, Hebei University of Technology, Tianjin, China; 6 School of Engineering, Saveetha University, Chennai, India; 7 Program in Materials Science, UFPI, Teresina, Brazil

**Keywords:** *Cyperus scariosus*, Valorization, Antacid, Phytochemicals, Environmental sciences, Chemistry

## Abstract

Agro-waste is the outcome of the under-utilization of bioresources and a lack of knowledge to re-use this waste in proper ways or a circular economy approach. In the Indian medicinal system, the root of *Cyperus scariosus* (CS) is used at a large scale due to their vital medicinal properties. Unfortunately, the aerial part of CS is treated as agro-waste and is an under-utilized bioresource. Due to a lack of knowledge, CS is treated as a weed. This present study is the first ever attempt to explore CS leaves as medicinally and a nutrient rich source. To determine the food and nutritional values of the neglected part of *Cyperus scariosus* R.Br. (CS), i.e. CS leaves, phytochemicals and metal ions of CS were quantified by newly developed HPLC and ICPOES-based methods. The content of the phytochemicals observed in HPLC analysis for caffeic acid, catechin, epicatechin, trans-p-coumaric acid, and trans-ferulic acid was 10.51, 276.15, 279.09, 70.53, and 36.83 µg/g, respectively. In GC–MS/MS analysis, fatty acids including linolenic acid, phytol, palmitic acid, etc. were identified. In ICPOES analysis, the significant content of Na, K, Ca, Cu, Fe, Mg, Mn, and Zn was observed. The TPC and TFC of the CS leaves was 17.933 mg GAE eq./g and 130.767 mg QCE eq./g along with an IC_50_ value of 2.78 mg/mL in the DPPH assay and better antacid activity was measured than the standard (CaCO_3_). The methanolic extract of CS leaves showed anti-microbial activity against *Staphylococcus aureus* (15 ± 2 mm), *Pseudomonas aeruginosa* (12 ± 2 mm) and *Escherichia coli* (10 ± 2 mm). In silico studies confirmed the in vitro results obtained from the antioxidant, antiacid, and anti-microbial studies. In addition, in silico studies revealed the anti-cancerous and anti-inflammatory potential of the CS leaves. This study, thus, demonstrated the medicinal significance of the under-utilized part of CS and the conversion of agro-waste into mankind activity as a pharmaceutical potent material. Consequently, the present study highlighted that CS leaves have medicinal importance with good nutritional utility and have a large potential in the pharmaceutical industry along with improving bio-valorization and the environment.

## Introduction

Agro-waste is the result of the under-utilization of bioresources and a lack of knowledge to re-use this waste in proper ways or during a circular economy approach^1^. The valorization of renewable resources like agro-waste can ameliorate environmental issues raised due to the transgression of mankind with the environment and that the world can overcome environmental issues through a circular economy concept^2^. Agro-industrial processes including landfills and open farming fields generate a huge quantity of diverse by-products that create environmental stress^3^. Agro-waste is rich of organic content and sources of bioactive compounds that exhibit medicinal and environmental cleaning properties^3^. For example, the agro-waste of rice contains beneficial phytochemicals like phenolic, flavonoids, and vitamins having medicinal properties^4^.

Agro-wastes are reported as a source of biogenic nano-materials^5^. The agro-waste can be utilized as packaging materials to enhance the shelf-life of food and meat products, and these packaging materials are biodegradable having anti-microbial properties^1^. Agro-waste materials and plants grown from agro-waste have shown a high yield of phenolic compounds and vitamin C^6,7^. The underutilized parts of herbs are expected to be a rich source of phytochemicals including antioxidant phenolics^8–10^. These phenolic compounds are well known for their broad-spectrum of medicinal properties including cardiovascular, anti-cancerous, anti-inflammatory, immunomodulatory, anti-aging, etc.^11–14^. There are selected reports on the utilization of agro-waste of herbs for the production of antioxidant phenolics, and which need to be further explored^11–13^.

*Cyperus scariosus* R.Br. (CS) or Nagarmotha belongs to the family Cyperaceae. It is a perennial herbaceous plant grown as a weed^15^. The detailed taxonomy and distributions are mentioned under supplementary data. CS is found around rivers, and waterfalls, especially found in damp or marshy regions as well as along coastal areas^16^. CS is widely distributed in the India, Australia, Malesia, China, South Africa, and the Pacific Islands^15–18^. The nuts of CS have dietary and medicinal applications. In India, CS is known as ‘Nagarmotha’ which is commonly known as “Nut grass”. According to Ayurveda, Nagarmotha rhizomes help improve digestion. Nagarmotha oil is an effective home remedy for managing stomach disorders due to its antispasmodic and carminative properties^15–18^. In the Indian Medicinal System, CS rhizomes are known for various biological activities including cordial, tonic, vermifuge, diuretic, diaphoretic, anti-diabetes, hepatoprotective activity, anti-inflammatory, hypotensive, spasmolytic, antioxidant and as a desiccant^19^. CS rhizomes are used to treat a variety of diseases including diarrhoea, epilepsy, gonorrhea, syphilis and liver disorders^19,20^. CS leaves are less explored where anti-nociceptive and anti-hyperglycemic activities are reported^16^. The combination of Boswellia serrata resin and roots/rhizomes of CS have shown significant effects on stress urinary incontinence^21^. Various phytoconstituents have been isolated from the rhizome part of CS and have been reported to include: 6-O-β-d-glucopyranosyl-O-α-l-rhamnopyranoside, leptosidin-6-O-[β-dxylopyranosyl (14)-β-d-arabinoside, stigmasta-5, and 24-(28)-diene-3 β-O-α-l-rhamnopyranosyl-O-β-d-aabinopyranoside as glycosides. The aroma of CS rhizomes or the root is due to the presence of volatile nitrogenous compounds like epi-guaipyridine, guaia-9,11-dienpyridine, and cananodine^22^.

The root and rhizome of CS plants have been extensively studied for medicinal and pharmaceutical studies, but none have studied the CS leaf as found in the literature. The CS leaves are treated as agro-waste and uprooted from the farming field due to a lack of knowledge. Overall, there is still no evidence of pharmacognostical evaluation, phytochemical, phenolics, antimicrobial and antioxidant properties of the CS leaves, which was the aim of the present study.

CS leaves are under-utilized and treated as agro-waste so there is a need to explore the agro-waste of CS leaves as a potent source for medicinal or nutritional values. There was a five-fold aim of the present study to explore the CS leaf, including to provide: (1) a detailed pharmacognostical evaluation of the CS leaf, (2) a phytochemical analysis and development of the HPLC method for their quantification, (3) an essential metal ion quantification of CS leaves for food and medicinal applications, (4) in vitro antacid, antioxidant and anti-microbial activities, and (5) in silico anti-cancer, anti-inflammatory, antacid, antioxidant and anti-microbial information.

## Results

### Pharmacognostic evaluation

**Figure 1:**
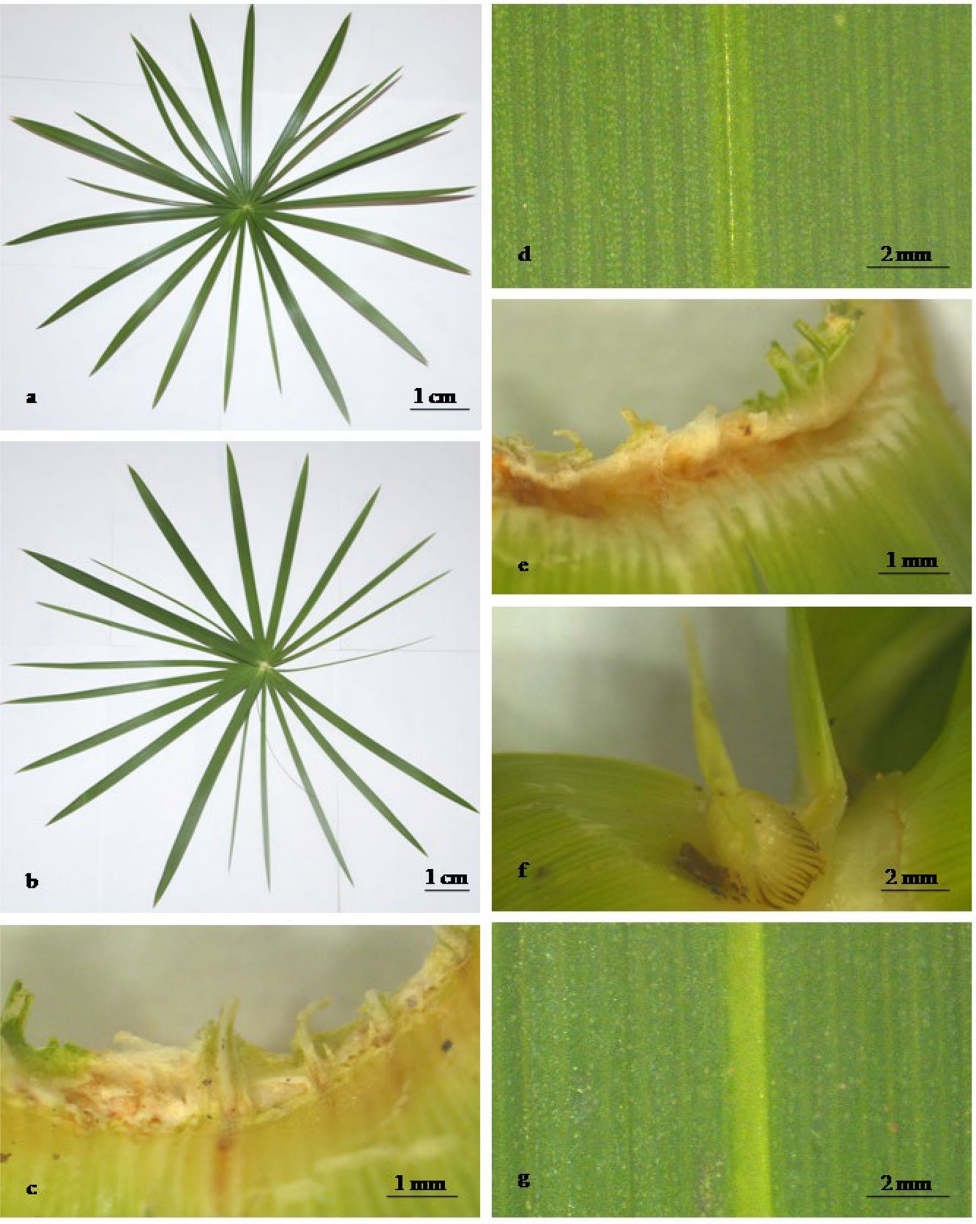
Leaf of (CS) *Cyperus scariosus* R. Br. (**a**) Ventral leaf, (**b**) dorsal leaf (**c** basal portion of the lower surface, **d** upper surface, **e** basal portion of the upper surface, **f** inflorescence axis, and **g** lower surface using stereo microscope LEICA S9D and EC4 camera).

The pharmacognosy analysis of CS leaf’s is unexplored, so detailed study is reported. The pharmacognosy analysis is the core study of any herbal material for the complete authentication and to avoid the adulteration^23^. The leaves of CS were alternate and tristichous and ligules were absent. Basal leaves were reduced to the sheath. Upper leaves were up to half the stem length, and the inflorescence axis was present at the base of the upper leaves. The leaf was linear in shape, with a sharp acute apex and was the widest at the base which was gradually decreasing, and the entire margin (Fig. 1a–g). The midrib was elevated at the lower surface while sunken at the upper surface. The upper surface was glossy and glabrous while the lower surface was minutely scabrous. The leaf venation was parallel. The leaf surfaces were green colored. The leaf had a characteristic odor (Fig. 1). Histochemical analysis of the CS leaves was evaluated and is shown in Table 1.

**Table 1:** Histochemical analysis of CS leaf (− negative, + positive).

Test	Chemical used	Observation if present	Plant part (leaf)
Aleurone grains	Iodine solution	Yellow color	**−**
Alkaloids	Dragendroff’s solution	Reddish brown ppt	+
Calcium carbonate	Acetic acid	Insoluble	−
Calcium oxalate	Acetic acid or HCl	Dissolve with effervescence	+
Cellulose	Iodine solution	Yellow color	+
Chitin	Sudan red III	Red	−
Cutin	Sudan red III, mount in glycerine and warm slightly	Red	−
Fixed oil and fats	Alcohol (90%)	Insoluble	−
Lignin	Phloroglucinol + Conc. HCl	Red- pink	+
Mucilage	Ruthenium red	Pink	−
Pectin	2% NaOH solution	Gelatinous ppt, on boiling becomes white	−
Resins	Sudan red III	Red	−
Starch	Iodine solution	Blue	+
Suberin	Iodine solution + sulphuric acid	Deep brown	+
Phenolics	Dil. Ferric chloride solution	Bluish black or green colour	+
Volatile oil	Alcohol (90%)	Soluble	+

**Figure 2:**
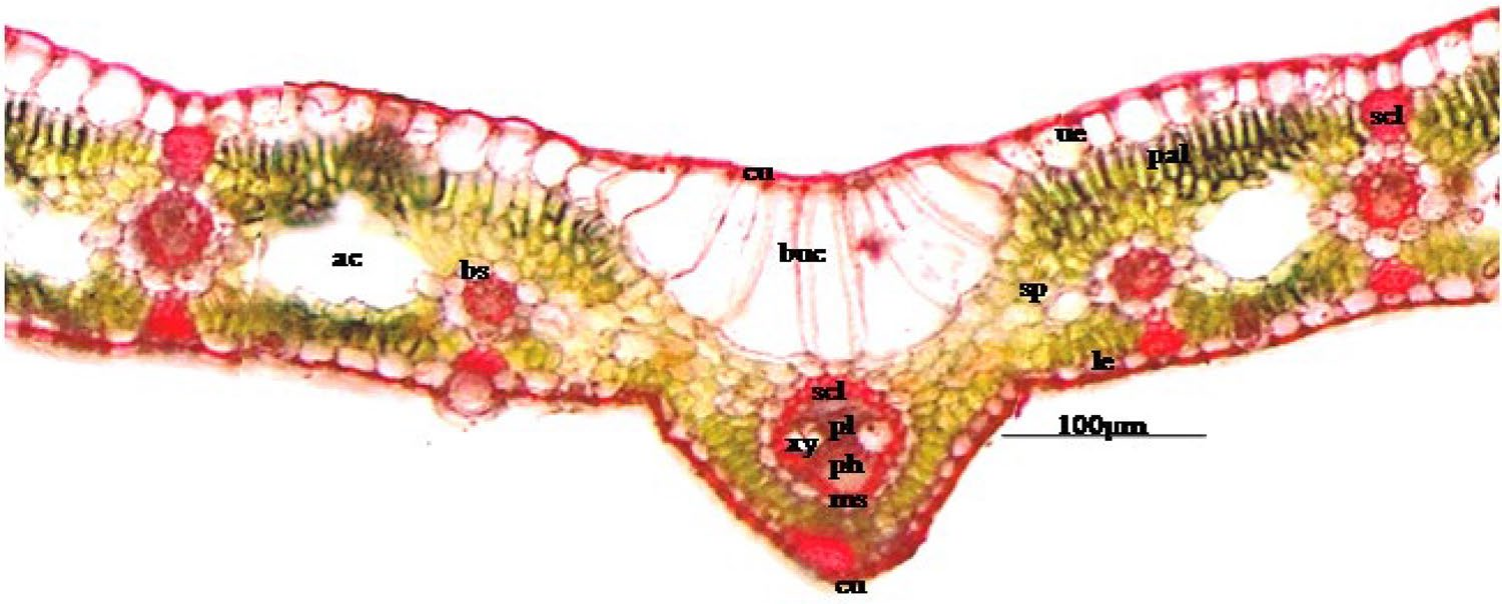
TS of leaf CS leaf at ×10 × ×10 using an Olympus microscope BX 43 and LC camera 30 [cuticle (cu), upper epidermis (ue), mesophyll (mes), palisade (pal), bulliform cells (buc), air cavity (ac), bundle sheath (bs), spongy parenchyma (sp), sclerenchyma (scl), lower epidermis (le), protoxylem lacuna (pl), xylem (xy), phloem (ph), and mestome sheath (ms)].

The TS of the dorsiventral leaf of CS showed a midrib composed of a slight depression on the upper surface and elevation at the lower surface covered with thick cuticle (cu) (Fig. 2). The upper epidermis (ue) was modified into irregularly barrel-shaped, longitudinally elongated, and larger bulliform cells (bc). Below bulliform cells, irregularly polygonal spongy cells were present which surrounded the vascular bundle. The barrel-shaped palisade cells were occurring below the upper epidermis and above the lower epidermis. Vascular bundles were conjoint, collaterally closed. The vascular bundle was composed of sclerenchyma, a bundle sheath, a mestome sheath, a protoxylem lacuna, xylem and phloem. Numerous vascular bundles were smaller as compared to a larger median vascular bundle. Sclerenchymatous bundle sheath extensions occurred in patches below and/or above the vascular bundle (Fig. 2). A bundle sheath was composed of compactly arranged, parenchymatous cells. A single-layered, lignified mestome sheath was seen near the bundle sheath cells. The xylem lied towards the upper epidermis, made up of thick-walled vessels, tracheids and xylem parenchyma. The phloem lied toward the lower epidermis and consisted of thin-walled, non-lignified cells. Each vascular bundle had two large metaxylem vessels present toward the lower surface and a protoxylem lacuna toward the upper surface (Fig. 2).

The TS of a dorsiventral leaf of CS showed cuticularized lamina comprised of the upper epidermis, mesophyll cells, vascular bundle and lower epidermis (Fig. 2). The upper epidermis (ue) was composed of compactly arranged, sub-rectangular parenchymatous cells which were covered with a cuticle. The mesophyll cells (mes) were differentiated into palisade cells and spongy parenchymatous cells. Two to three layers of isobilateral, barrel-shaped palisade cells deposited with chlorophyll were seen, and irregularly polygonal spongy cells were sandwiched between them which was deposited with chlorophyll. The vascular bundles were smaller than the midrib (Fig. 2). Sclerenchymatous bundle sheath extensions occurred in patches toward the inner side of both the upper and lower epidermis. The stomatal number was 53–56–59 per sq mm for the upper epidermis, and 145–205–265 per sq mm for lower epidermis; the stomatal index was 6.6002–6.7995–6.9988 per sq mm for the upper epidermis, and 14.6464–15.6460–16.6457 per sq mm for the lower epidermis (Fig. 2).

**Figure 3:**
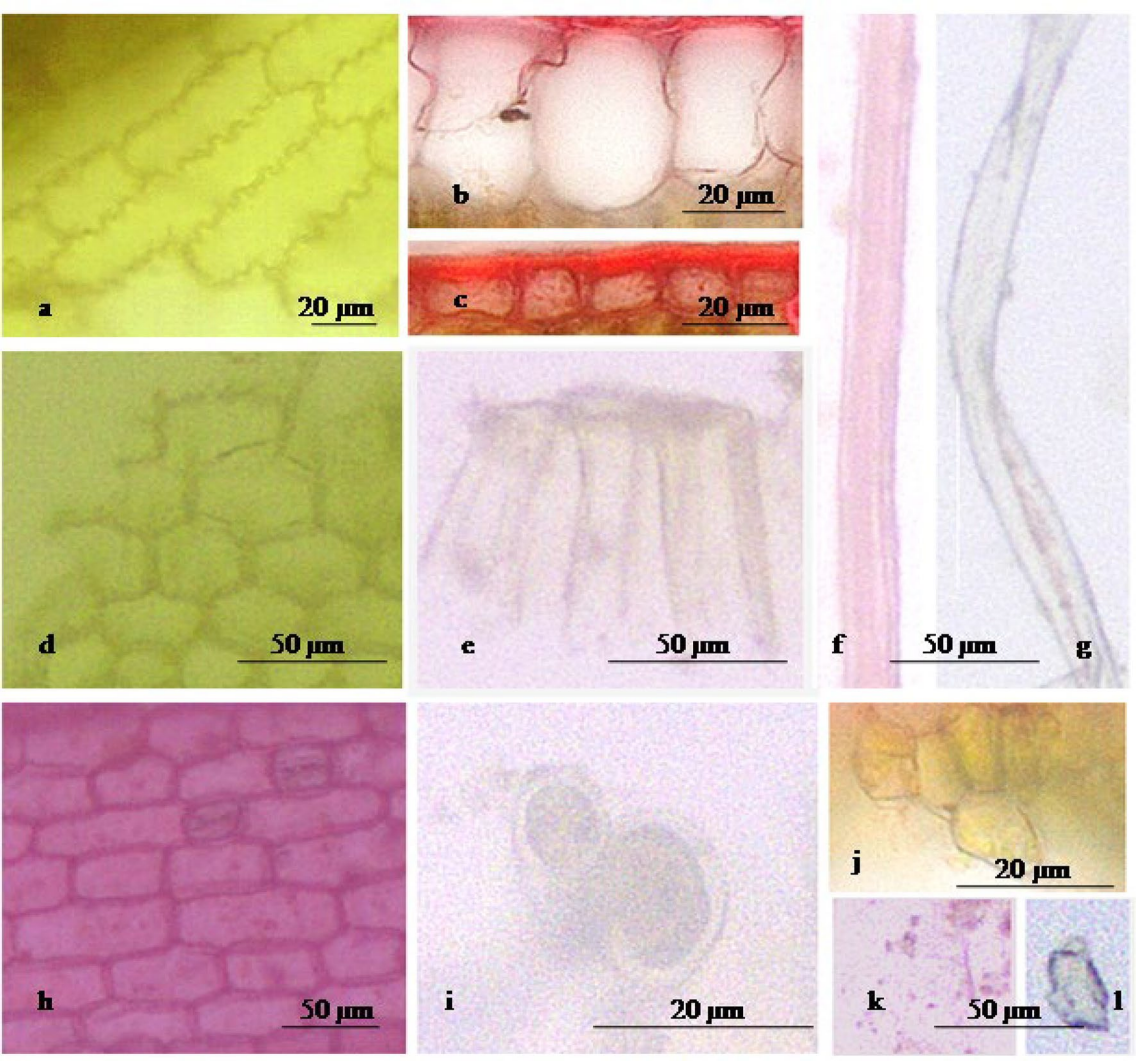
Powder microscopy of CS leaf at ×10 × ×10 and ×40 × ×10 using an Olympus microscope BX 43 and LC camera 30 [**a** epidermis in surface view, **b** upper epidermis in sectional view, **c** lower epidermis in sectional view, **d** parenchymatous cells in surface view, **e** bulliform cells in sectional view, **f** lignified fiber, **g** non-lignified fiber, **h** epidermis with stomata in surface view, **i** mesophyll cells in surface view, **j** mesophyll cells in sectional view, **k** starch grains, and **l** prismatic crystal of calcium oxalate].

Various characteristics were shown in the powder microscopy of the leaf of CS (Fig. 3a–l). The fragment of a rectangular, wavy walled with sharp angle and gramineous stomata of the epidermis in surface view was seen. The upper epidermis had sub-rectangular, compactly arranged parenchymatous cells in a sectional view while the lower epidermis had thick-walled, quadrangular parenchymatous cells in a sectional view. Hexagonal parenchymatous cells were seen in the surface view. Elongated, quadrangular bulliform cells in sectional view were present. In the surface view, oval-shaped mesophyll cells were seen while in the sectional view it was polygonal shaped. Other characteristics were found in powder microscopy, including a fragment of elongated lignified and non-lignified fiber, starch grains and prismatic crystal of calcium oxalate.

### HPLC–DAD analysis

Optimization of the chromatographic conditions incorporated the use of water, acetonitrile, methanol, and water (0.1% formic acid) in the mobile phase and changes in the flow rate and gradient (Table 2). The optimized chromatographic conditions produced the best results in terms of peak resolution, symmetry, and sharpness. The validation of the developed method was also carried out as per ICH guidelines and the results for method validation parameters are mentioned in Table 2 and the Supplementary data (Figure S1). The calibration curves for the analyzed phytochemical standards exhibit great linearity with a determination coefficient R^2^ > 0.999.

**Table 2:** HPLC-based method validation data for CS leaf extracts and marker compounds.

Compound	λ_max_ (nm)	Linearity Equation	Determination Coefficient (R^2^)	Linearity range (ng/µL)	LOD (ng/µL)	LOQ (ng/µL)
Linearity and sensitivity						
Caffeic acid	326	y = 61.84x − 27.62	0.9998	1–60	0.32	0.98
Catechin	280	y = 5.23x − 3.73	0.9998	5–60	1.63	4.95
Epicatechin	280	y = 4.61x − 3.84	0.9997	5–60	1.64	4.97
Trans-p-coumaric acid	326	y = 63.20x − 27.91	0.9998	1–60	0.30	0.91
Trans-ferulic acid	326	y = 60.17x − 25.69	0.9998	1–60	0.33	0.99

Compound	Amount (ng/µL)	Precision (% RSD)				
		Intra-day (n = 3)		Inter-day (n = 3)		
		RT (min)	Peak area	RT (min)	Peak area	
Precision						
Caffeic acid	10	0.14	0.29	0.70	2.31	
	20	0.03	0.75	0.69	1.22	
	40	0.14	0.62	0.52	0.24	
Catechin	10	0.17	1.76	1.09	2.26	
	20	0.04	1.74	1.08	2.40	
	40	0.18	0.42	0.84	0.32	
Epicatechin	10	0.15	1.71	0.85	2.16	
	20	0.02	1.33	0.86	2.04	
	40	0.09	0.86	0.67	1.01	
Trans-p-coumaric acid	10	0.14	0.26	0.53	1.48	
	20	0.02	0.68	0.52	0.80	
	40	0.06	0.67	0.39	0.21	
Trans-ferulic acid	10	0.08	0.22	0.35	1.36	
	20	0.02	0.48	0.22	0.15	
	40	0.09	0.61	0.27	0.44	

Compound	% Recovery					
	Spike level-1	Spike level-2	Spike level-3			
Accuracy						
Caffeic acid	104.32	106.51	103.48			
Catechin	96.21	99.63	97.52			
Epicatechin	98.73	96.86	94.37			
Trans-p-coumaric acid	103.26	104.27	106.77			
Trans-ferulic acid	104.75	102.83	103.28			

**Figure 4:**
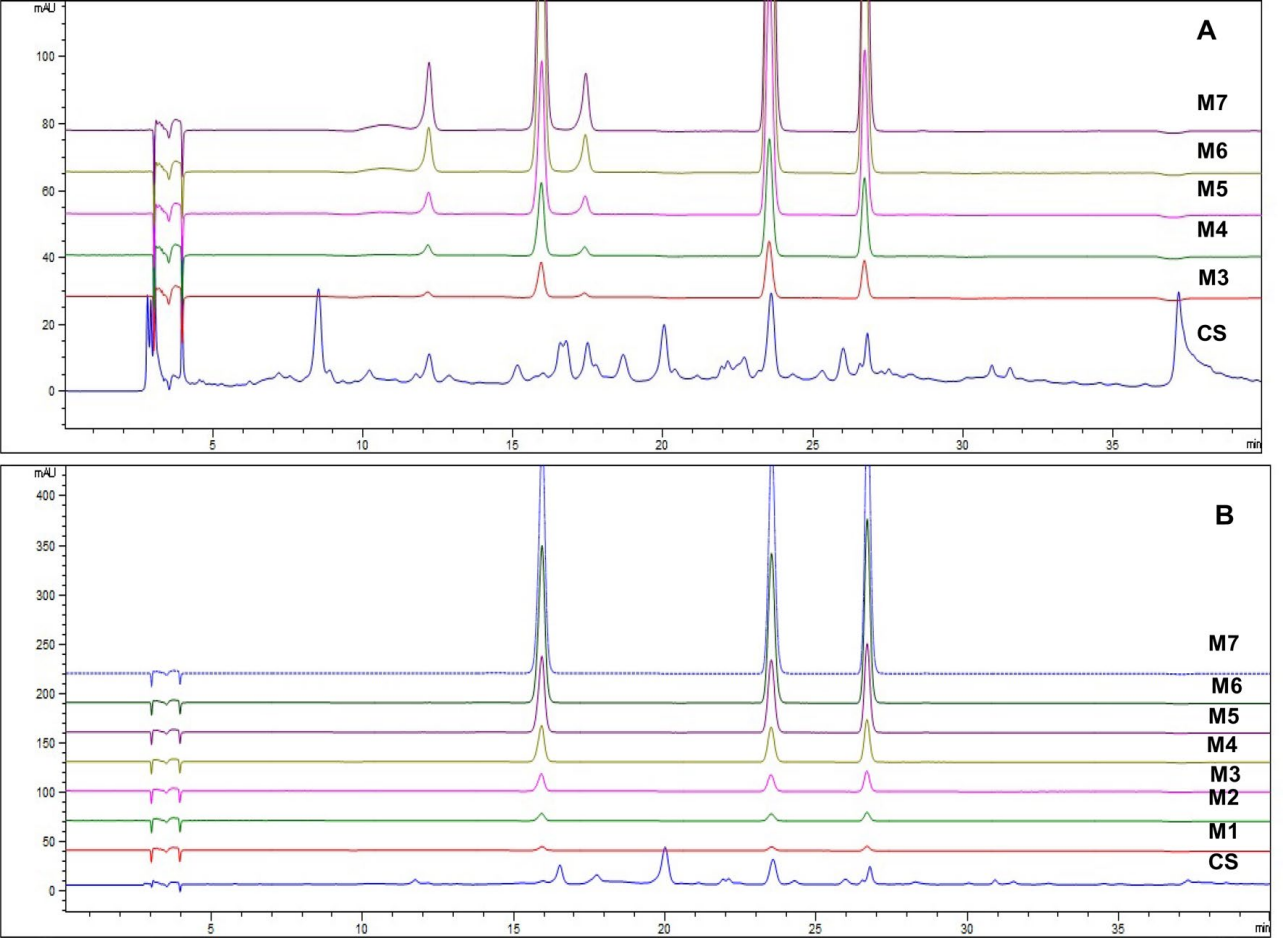
HPLC chromatograms of standards and sample extracts under 280 nm (**A**) and 326 nm (**B**) of CS leaf.

The corresponding chromatograms along with the chromatograms of sample extracts are demonstrated in Fig. 4 and the respective calibration curves are shown in the Supplementary data (Figure S1). In HPLC–DAD analysis, the observed retention factors (Rt) for the caffeic acid, catechin, epicatechin, trans-p-coumaric acid, and trans-ferulic acid were 15.97, 12.18, 17.46, 23.58, and 26.79 min, respectively (Fig. 4). The content of different phytochemicals in the CS leaf methanolic extracts evaluated by HPLC–DAD method for caffeic acid, catechin, epicatechin, trans-p-coumaric acid, and trans-ferulic acid were 10.51 (± 1.04), 276.15 (± 14.23), 279.09 (± 15.11), 70.53 (± 8.77), and 36.83 (± 3.44) µg/g, respectively. The best separation of phytochemicals viz. caffeic acid, catechin, epicatechin, trans-p-coumaric acid, and trans-ferulic acid without compromising resolution was achieved with column C18 (4.6 × 250 mm, 5µm particle size) and the conditions mentioned in the “Material and method” section.

### GC–MS/MS analysis

**Figure 5:**
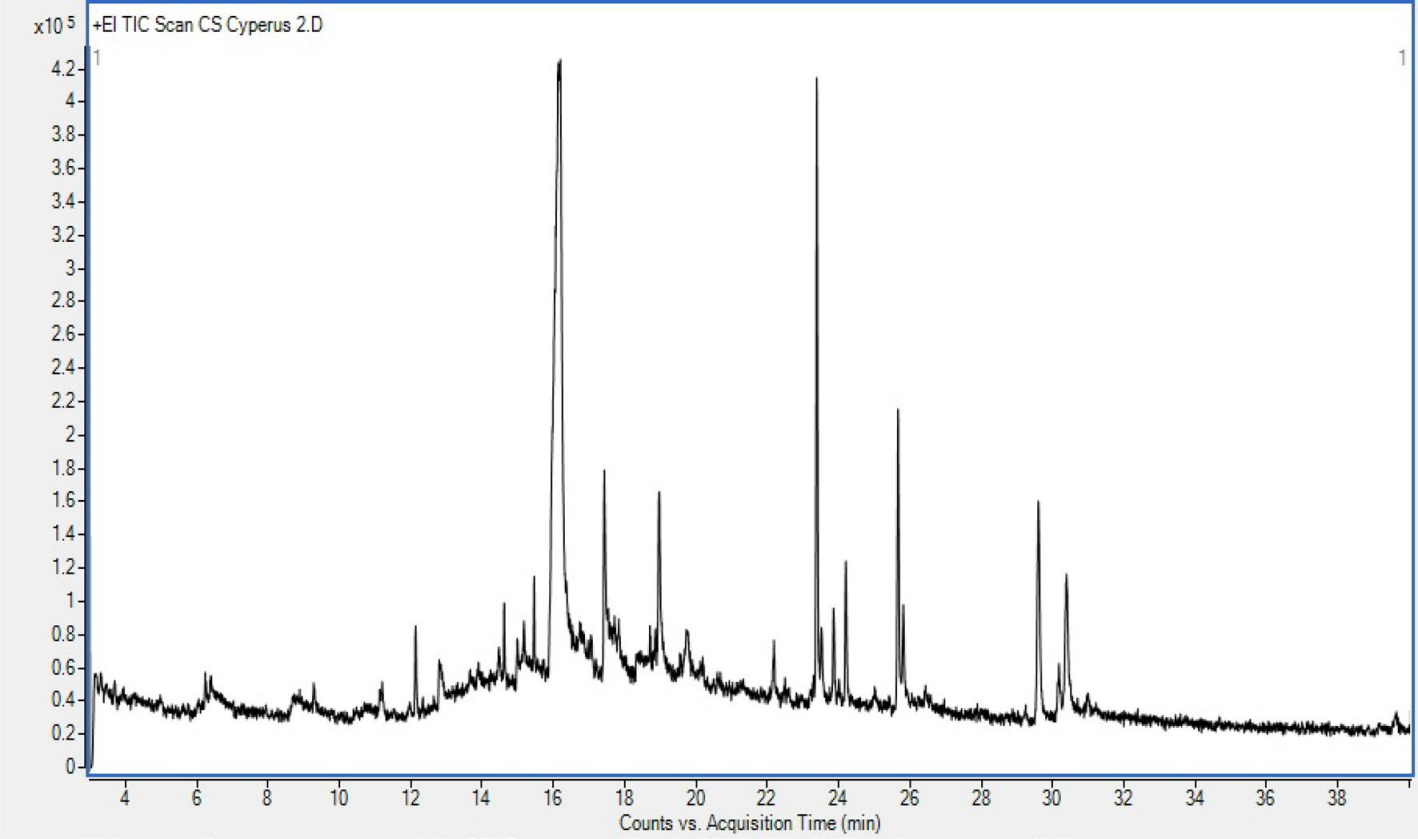
GC–MS chromatogram of the methanolic extract of CS leaf.

The GC–MS/MS chromatogram of methanolic extract of CS leave has exhibited thirty phytochemicals along with sixteen major phytochemicals (Fig. 5). The phytochemicals viz. stearyl vinyl ether, 6-butyl-1-nitro-1-cyclohexene, 3,7,11-trimethyl-1-dodecanol, melezitose, methyl 4-O-acetyl-2,3,6-tri-O-ethyl-α-d-galactopyranoside, β-hydroxydodecanoic acid, β-lactose, 4-hydroxy-3,5-dimethoxybenzohydrazide, 3,7,11,15-tetramethyl-2-hexadecen-1-ol, 1-dodecanol, 3,7,11-trimethyl-17-octadecynoic acid, 13-heptadecyn-1-ol, palmitic acid, butyl octyl phthalate, phytol, and linolenic acid were identified at 12.134, 15.172, 15.457, 16.191, 17.428, 18.959, 19.727, 22.180, 23.383, 23.509, 23.852, 24.195, 25.663, 25.808, 29.594, and 30.387 min, respectively. The observed percentage peak area for these 16 phytochemicals were 1.31, 1.43, 0.90, 52.34, 4.68, 4.12, 1.71, 1.03, 9.77, 1.66, 1.63, 2.37, 4.93, 1.25, 6.43, and 4.45%, respectively. In GC–MS/MS analysis, the maximum dominance was of melezitose (52.34%) followed by 3,7,11,15-tetramethyl-2-hexadecen-1-ol (9.77%), phytol (6.43%), palmitic acid (4.93%), methyl 4-O-acetyl-2,3,6-tri-O-ethyl-α-d-galactopyranoside (4.68%), linolenic acid (4.45%) and β-hydroxydodecanoic acid (4.12%). There are more than 10 hydrocarbons of CS leaves that have been identified using GC–MS/MS analysis.

### Elemental analysis

The elemental analysis of CS leaves indicated the presence of essential elements in significant amounts and heavy metals below permissible limits. The amount of heavy metal ions viz. the wavelength for analysis, linearity range, calibration equations, and determination coefficients are mentioned in Table 3. The calibration curves for respective elements are also shown in the Supplementary data (Table 3; Figure S2). The observed amount of As, Cd, Hg, and Pb were 0.25 (± 0.05), 0.07 (± 0.01), 0.14 (± 0.03), and 3.81 (± 0.34) mg/kg, respectively. The CS leaves were found to be a good source of essential metal ions viz. Na, K, Ca, Cu, Fe, Mg, Mn, and Zn which were at 566.57 (± 23.12), 15,195.96 (± 88.21), 4774.27 (± 43.88), 16.78 (± 3.22), 137.67 (± 6.66), 2463.76 (± 29.24), 57.81 (± 4.12), and 60.25 (± 3.33) mg/kg, respectively.

**Table 3:** Calibration curve equations and linearity of elemental standards.

Element	Wavelength (nm)	Linearity range (ppb)	Linearity equation	Determination coefficient (R^2^)
Na	589.592	100–10,000	y = 191.85 x + 10,880.11	0.9992
K	769.897	100–10,000	y = 42.12 x + 11,728.17	0.9994
Ca	396.847	100–10,000	y = 2945.28 x + 103,302.83	0.9993
Cu	324.754	100–10,000	y = 48.49 x + 1205.95	0.9999
Fe	259.94	100–10,000	y = 26.79 x + 45.15	0.9999
Mg	279.553	100–10,000	y = 955.89 x + 7055.25	0.9993
Mn	259.372	100–10,000	y = 176.99 x + 90.92	0.9999
Zn	334.502	100–10,000	y = 2.60 x + 8.66	1.0000
As	188.980	30–240	y = 2.71x + 8.35	0.9999
Cd	226.502	3–24	y = 107.94 x + 10.35	1.0000
Hg	194.164	30–240	y = 7.67 x + 15.45	0.9999
Pb	220.353	200–1600	y = 0.53 x − 1.99	0.9998

### Antioxidant activity

The antioxidants present in the plants exhibit a wide range of biological activities. Taking into account the relationship between phenolics and flavonoids and the antioxidant activity of botanicals, TPC, TFC and the antioxidant activity (DPPH radical scavenging activity) of the CS leaf extract, we also evaluated and determined the calibration curves for the standards, gallic acid (y = 0.0010x − 0.0218; r^2^ = 0.99) and quercetin (y = 0.0002x + 0.0011; r^2^ = 0.99), as shown in the Supplementary data (Figure S3). The results for TPC and TFC values are expressed in terms of mg GAE/g wt, mg QCE/g wt, respectively, and the results for DPPH radical scavenging activity are expressed in terms of IC_50_ values and TEAC values. The observed TPC and TFC for CS leaves were 17.933 (± 1.21) mg GAE eq./g of wt and 130.767 (± 5.67) mg QCE eq./g of wt, respectively. The DPPH assay depicted the strong antioxidant activity of CS leaf extracts with an IC50 value of 2.78 (± 0.21) mg/mL and with a TEAC value of 0.0102 (± 0.0012). The significant antioxidant capacity of CS leaves is linked with the phytochemicals identified in HPLC and GC–MS/MS analysis.

### Antacid activity

**Figure 6:**
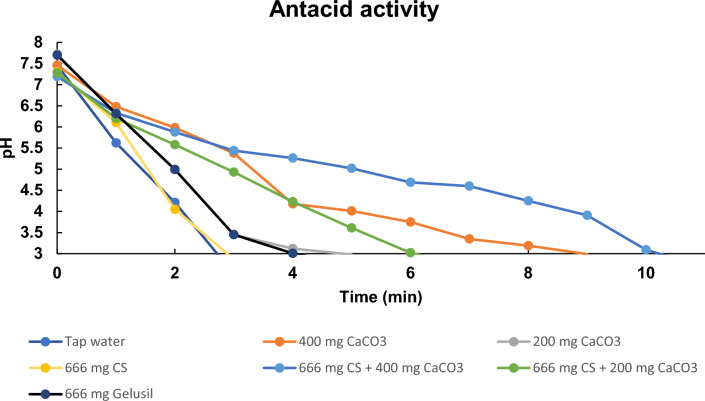
Antacid profiles of tap water, CaCO_3_ and different combinations with CS leaf powder.

The antacid profiles of tap water, CaCO_3_, different combinations of CS with CaCO_3_, and an antacid dug (Gelusil) was analyzed and reported (Fig. 6). The observed antacid activity trend was 666 mg CS + 400 mg CaCO_3_ > 400 mg CaCO_3_ > 666 mg CS + 200 mg CaCO_3_ > 200 mg CaCO_3_ > 666 mg Gelusil > tap water. It has been observed that 666 mg CS + 400 mg CaCO_3_ possessed more gastric resistance time, i.e. antacid action (10.2 ± 0.43 min) as compared to the standard 400 mg CaCO_3_ (8.5 ± 0.45 min) and a market drug of 666 mg Gelusil (4.0 ± 0.05 min). Thus, the present invitro study confirmed the folk claim for the CS leaf as a gastric pain relaxant.

### Antimicrobial analysis

**Figure 7:**
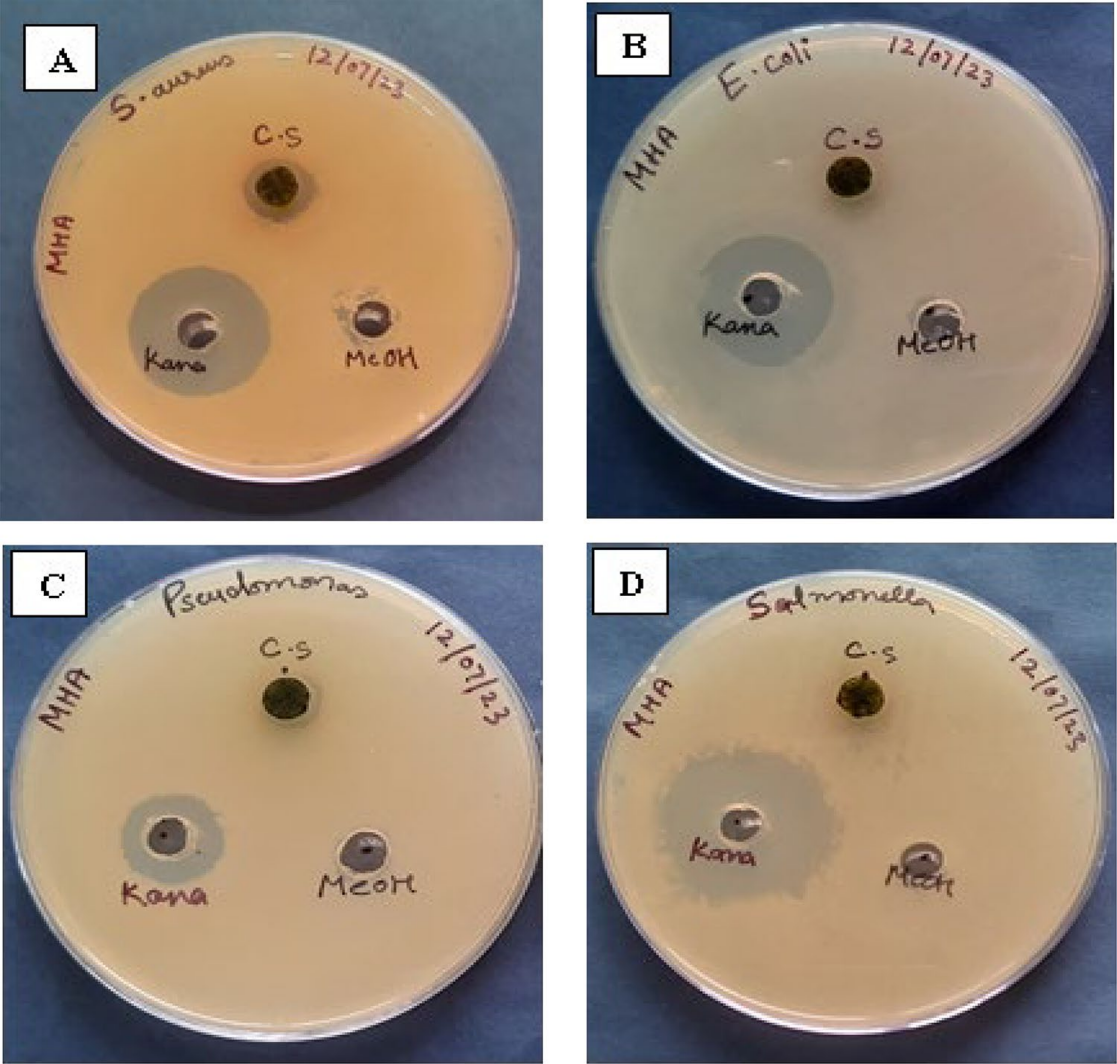
Antibacterial activity of *Cyperus scariosus* (C.S) leaf extract against (**A**) *Staphylococcus aureus* (MTCC No. 1430^T^), (**B**) *Escherichia coli* (MTCC No. 1885), (**C**) *Pseudomonas aeruginosa* (MTCC No. 424) and (**D**) *Salmonella typhi* (MTCC No. 733).

The antibacterial activity of the CS leaves extracts against the selected gram positive and gram-negative microorganisms was analyzed by comparing the zone of inhibition of bacteria by the test extract with the standard kanamycin. The methanolic extract (100 mg/mL) of CS leaves showed zone of inhibition trends as: *Staphylococcus aureus* (15 ± 2 mm) > *Pseudomonas aeruginosa* (12 ± 2 mm) > *Escherichia coli* (10 ± 2 mm) > *Salmonella typhi* (8 ± 2 mm) (Fig. 7). In comparison with standard kanamycin, methanolic extracts of CS leaves revealed a clear zone of inhibition against *Staphylococcus aureus.* In the case of *Pseudomonas aeruginosa* and *Escherichia coli*, a zone of inhibition was observed but less than *Staphylococcus aureus*. The methanolic extract of CS leaves showed poor inhibition against *Salmonella typhi* (8 ± 2 mm) (Fig. 7). There has been no antimicrobial study on CS leaves, so this study may help to convert agro-waste of CS leaves into a pharmaceutical potent raw antibacterial material, although clearly more and in-depth studies are needed to develop any formulation.

### Computational studies

Computational studies were performed to confirm or cross validate the prior in vitro studies demonstrating anti-microbial, antacid and antioxidant activities. Moreover, two additional activities (viz. anti-cancer and anti-inflammatory) were also analyzed in in silico studies. The detailed biological activities of the phytochemicals observed in present study through HPLC and GC–MS analyses is mentioned in Table 4.

**Table 4:** Biological applications of phytoconstituents CS leaf observed in HPLC and GC–MS study.

Compound	Applications	References
Caffeic acid	Antioxidant, anti-inflammatory and anticarcinogenic. Nutraceutical agent and Cosmetic agent	^24^
Catechin and Epicatechin	Anti-microbial, anti-viral, anti-inflammatory, anti-allergenic, and anti-cancer. Nutraceutical agent and Cosmetic agent	^25^
Trans-p-coumaric acid	Antioxidant, antimicrobial, anticancer, antiarthritic, anti-inflammatory, gout prevention, anti-diabetic, anti-melanogenic, skin regeneration, gastroprotective, anti-ulcer, cardioprotective, hepatoprotective, reno-protective, bone formation, anti-angiogenic and anti-platelet	^26^
trans-ferulic acid	Antioxidant, antimicrobial, anti-inflammatory, anti-thrombosis, and anti-cancer activities	^27^
Melezitose	Nutraceutical agent and Masking agent in the food industry and a replacement of sugar additives	^28^
Phytols, Linolenic acid, Palmitic acid, n-hexadecanoic acid	Nutraceutical agent, anti-inflammatory, anticancer and anti-oxidant	^29,30^

**Figure 8:**
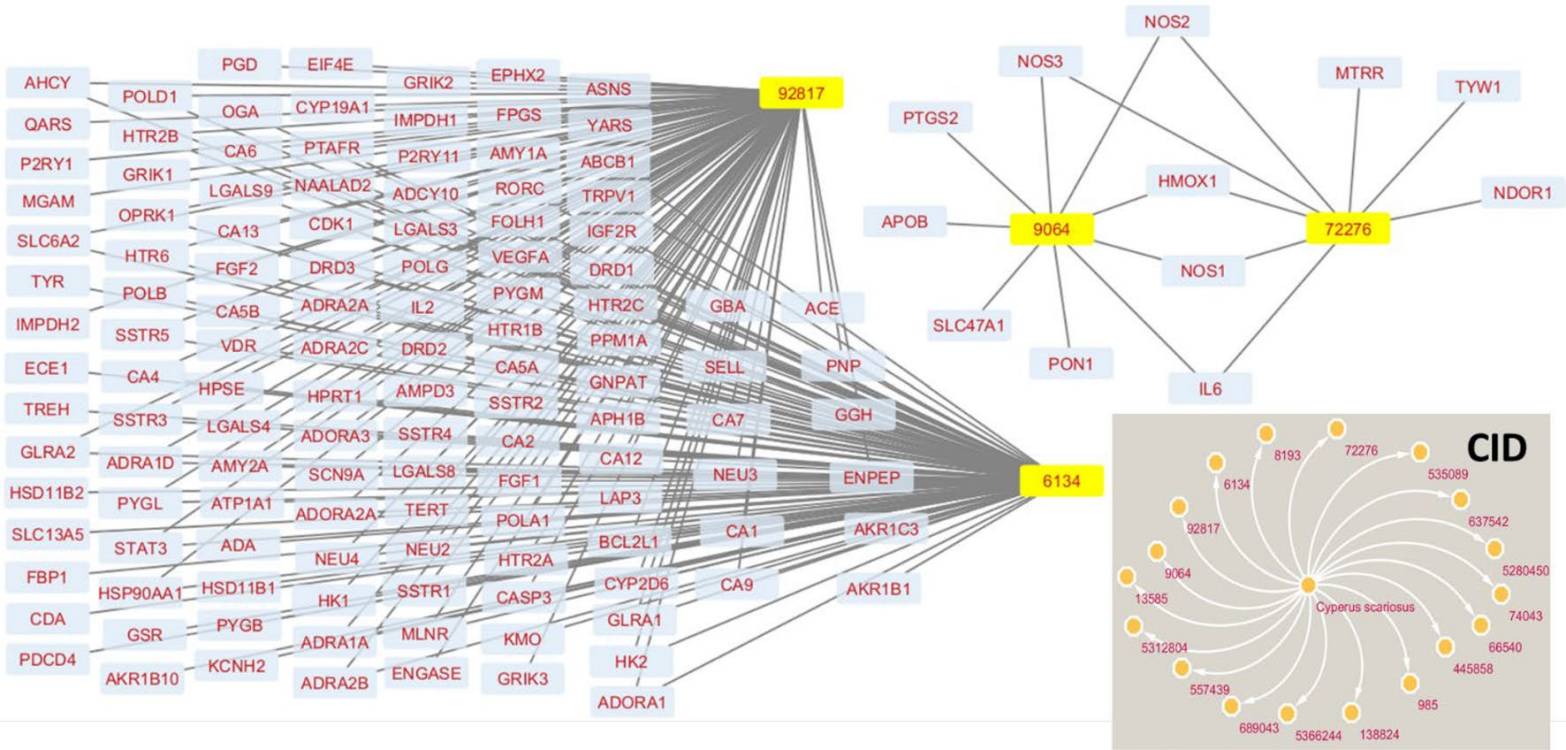
Pubchem Ids (CID) of selected compounds and four compounds have passed drug likeness filters.

**Figure 9:**
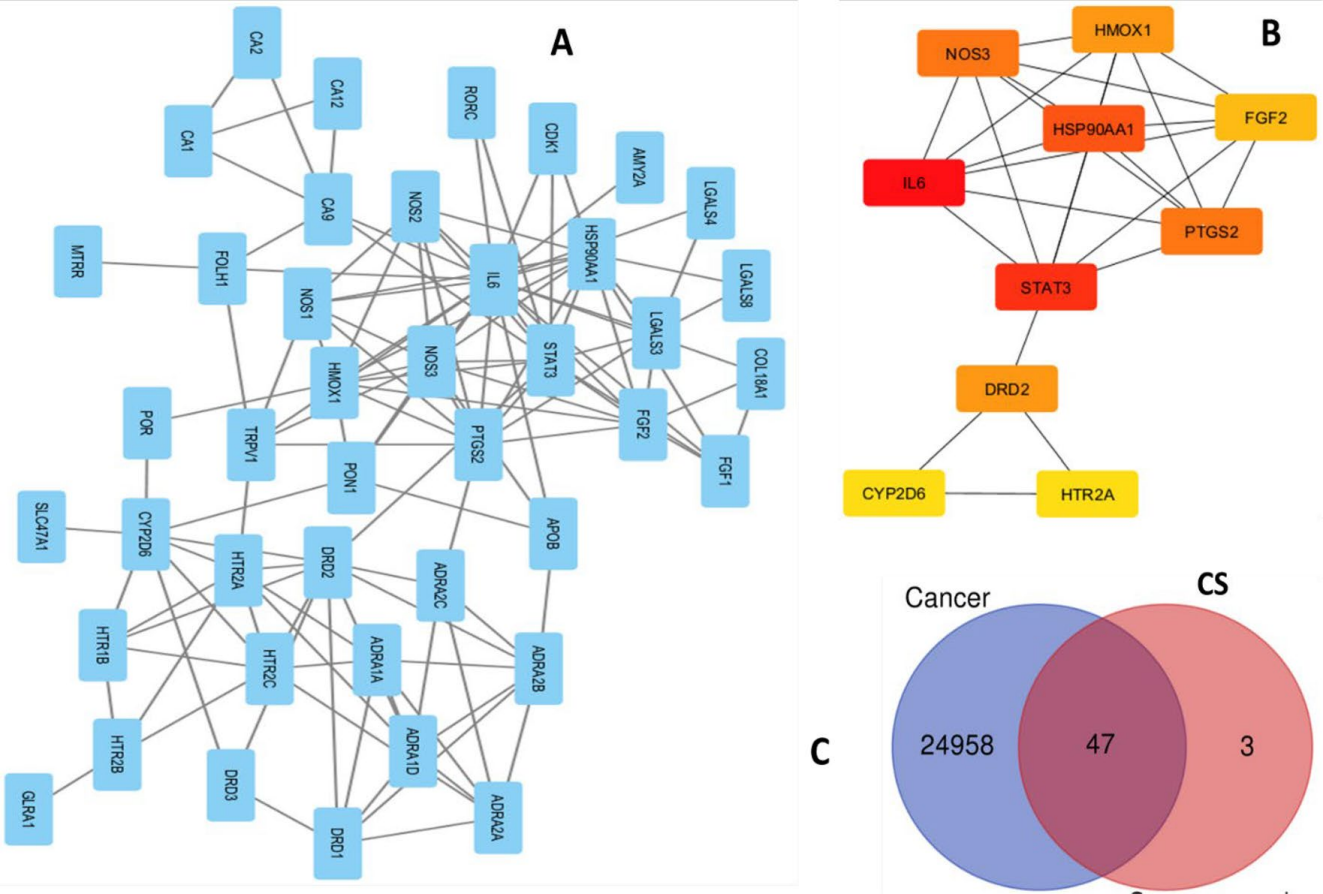
Interactions with various genes (**A**), top ten genes with the shortest path (**B**), Venn diagram represents the potential genes between the CS and cancer (**C**).

In network pharmacology study, the ligand names as observed through HPLC and GC–MS studies have been replaced with pubchem Ids (CID) of compounds. Out of 18 compounds, only four compounds having pubchem Ids 92,817 (Melezitose), 9064 (Catechin), 6134 β-lactose) and 72,276 (Epicatechin) passed the drug likeness filter (Fig. 8). Out of 25,000 cancer genes, 50 interacting target genes of CS, 47 were overlapping with the subset of cancer genes, indicating probable anticancer activity (Fig. 9). Out of them, the top ten genes with the shortest path based on degree and interacting with highest number of genes is also plotted (Fig. 9). The top ten in network ranked by degree method were as [name (Score or no. of interacting genes)]: IL6 (20), STAT3 (13), HSP90AA1 (12), NOS3 (11), PTGS2 (11), DRD2 (10), HMOX1 (10), FGF2 (9), CYP2D6 (8), and HTR2A (8) respectively (Fig. 9).

The Maestro v 13.1 ligand docking module was used to complete docking after producing the glide grid zip file and getting the ligands ready. Certain phytoconstituents of CS leaves are molecularly docked with greater accuracy using the enhanced precision (XP) module. As the degree of precision rises, the magnitude of the data collection reduces. In Maestro v 13.1, docking score, glide energy, and glide model value are provided as XP parameters^31,32^. The phytochemicals like caffeic acid, catechin, epicatechin, trans-p-coumaric acid, trans-ferulic acid, stearyl vinyl ether, 6-butyl-1-nitro-1-cyclohexene, 3,7,11-trimethyl-1-dodecanol, melezitose, methyl 4-O-acetyl-2,3,6-tri-O-ethyl-α-d-galactopyranoside, β-hydroxydodecanoic acid, β-lactose, 4-hydroxy-3,5-dimethoxybenzohydrazide, 3,7,11,15-tetramethyl-2-hexadecen-1-ol, 1-dodecanol, 3,7,11-trimethyl-17-octadecynoic acid, 13-heptadecyn-1-ol, palmitic acid, butyl octyl phthalate, phytol, and linolenic acid were examined in an in silico analysis along with five standard medications, including azathioprine, cimetidine, trolox, aspirin, and kanamycin. Table 4 exhibited that more than five phytoconstituent ligands exhibited higher docking scores and glide energies as compared to standards (azathioprine, cimetidine, trolox, aspirin, and kanamycin) (Table 5). The 2D and 3D ligand–protein interactions and chemical interactions of phytoconstituents along with standard medications are mentioned under Supplementary Figures S4–S9.

**Table 5:** Phytoconstituents of CS leaf docking screening in comparison to different standard drugs.

Compounds	Docking score (PDB ID: 2VCZ)	Docking score (PDB ID: 1G1T)	Docking score (PDB ID: 1OG5)	Docking score (PDB ID: 6BL4)	Docking score (PDB ID: 5X14)
Caffeic acid	**− 5.873**	**− 3.402**	**− 8.039**	**− 7.407**	**− 8.011**
Catechin	**− 7.823**	**− 6.181**	**− 6.576**	**− 10.541**	**− 7.964**
Epicatechin	**− 7.823**	**− 6.181**	**− 6.576**	**− 10.541**	**− 7.964**
Trans-p-coumaric acid	**− 5.15**	**− 2.778**	**− 6.234**	**− 6.408**	**− 6.308**
trans-ferulic acid	**− 6.389**	**− 3.554**	**− 6.075**	**− 6.887**	**− 7.859**
Stearyl vinyl ether	− 2.382	1.274	− 4.059	− 2.796	− 0.737
6-Butyl-1-nitro-1-cyclohexene	− 3.262	− 0.025	− 2.845	− 4.456	− 3.409
3,7,11-Trimethyl-1-dodecanol	− 1.1683	0.285	**− 5.316**	− 5.819	− 1.729
Melezitose	**− 11.811**	**− 5.038**	**− 10.671**	− **8.388**	**− 6.658**
Methyl 4-O-acetyl-2,3,6-tri-O-ethyl-α-d-galactopyranoside	− 4.107	− 2.282	− 0.053	− 2.323	− 1.956
β-Hydroxydodecanoic acid	− 3.085	− 1.064	**− 6.923**	− 5.297	− 2.688
β-Lactose	**− 6.259**	**− 4.494**	**− 7.528**	− 5.655	**− 6.118**
4-Hydroxy-3,5-dimethoxybenzohydrazide	− 4.456	**− 3.804**	**− 5.342**	**− 6.076**	**− 6.333**
3,7,11,15-Tetramethyl-2-hexadecen-1-ol,	− 1.953	0.048	− 3.855	**− 8.007**	− 1.3
1-Dodecanol	− 0.495	1.772	**− 5.03**	− 3.772	− 0.275
3,7,11-trimethyl-17-Octadecynoic acid	− 4.558	− 0.31	− 3.898	**− 9.048**	− 3.787
17-Octadecynoic acid	− 2.916	1.909	**− 6.516**	− 5.435	− 1.68
13-Heptadecyn-1-ol	− 1.842	1.495	**− 5.507**	− 4.97	− 0.777
Palmitic acid	− 1.669	1.27	**− 4.229**	− 5.042	− 2.849
Butyl octyl phthalate	− 3.916	− 2.129	**− 7.437**	**− 7.537**	− 2.431
Phytol	− 2.974	0.946	− 2.76	**− 7.101**	− 1.506
Linolenic acid	− 2.54	1.348	**− 5.915**	**− 7.195**	− 2.279
Standard drug	Azathioprine (anticancer) − 4.898	Cimetidine (antacid) − 2.569	Trolox (antioxidant) − 3.937	Aspirin (anti-inflammatory) − 5.997	Kanamycin (anti-microbial) − 5.723

Bold letter indicating significantly different results.

Overall, caffeic acid, catechin, epicatechin, trans-p-coumaric acid, trans-ferulic acid, melezitose, β-lactose and 4-hydroxy-3,5-dimethoxybenzohydrazide showed a good score as compared to standard drugs (w.r.t. anti-cancer, anti-inflammatory, antacid, antioxidant and antimicrobial studies). In antacid in silico analysis, all of the above-mentioned phytochemicals had a better score than the standard drug cimetidine. Similar trends were observed in the case of anti-microbial in silico studies. In comparison with the standard drug Trolox, except Phytol, 3,7,11-trimethyl-17-octadecynoic acid, 3,7,11,15-tetramethyl-2-hexadecen-1-ol, methyl 4-O-acetyl-2,3,6-tri-o-ethyl-α-d-galactopyranoside, stearyl vinyl ether and 6-butyl-1-nitro-1-cyclohexene, all other phytochemicals showed significant antioxidant potential. In addition to in vitro studies, additionally, anticancer and anti-inflammatory in silico studies were performed. For anti-inflammatory activity, the maximum phytochemicals including caffeic acid, catechin, epicatechin, trans-p-coumaric acid, trans-ferulic acid, melezitose, butyl octyl phthalate, phytol, linolenic acid, 3,7,11,15-tetramethyl-2-hexadecen-1-ol, 4-hydroxy-3,5-dimethoxybenzohydrazide, and 3,7,11-trimethyl-17-octadecynoic acid showed a better score than the standard drug aspirin. Caffeic acid, catechin, epicatechin, trans-p-coumaric acid, trans-ferulic acid, melezitose, and β-lactose showed significant anti-cancer potential as compared to the standard drug azathioprine (Table 5).

## Discussion

In literature, *Cyperus rotundus* Linn (*C. rotundus*) and *Cyperus rotundus* Linn are mentioned as same, but both are different herbs^20–22^. Pharmacognosy study has provided the detailed variance. In the present study, in comparison with the *Cyperus rotundus* Linn (*C. rotundus*) leaf, the CS leaf morphologically resembled the *C. rotundus* Linn leaf. There are specific characteristics that will help in distinguishing them including the basal leaves were sheathing while it was not like that in *C. rotundus.* Also, the top leaves formed a fan-shaped canopy while *C. rotundus* was not forming such a canopy which had few leaves. The leaves showed an acute apex while *C. rotundus* had acuminate^33^. Moreover, the upper leaves were up to half the stem length while it was shorter or longer than the stem length in *C. rotundus.* Many times, the plant resembled morphologically others and it was difficult to find plant species in the field. Hence, the specific morphological characteristics will help in the identification of the correct plant. In the transverse section, characteristic features of a CS leaf were compared with *C. rotundus*. The cuticle was present in CS while it was absent in *C. rotundus.* The leaf had an air cavity between each vascular bundle while *C. rotundus* had no air cavity. In the lamina region, the alternate vascular bundles and air cavity occured while vascular bundles were present in a continuous row into the *C. rotundus*. The mesophyll cells were differentiated into palisade and spongy parenchymatous cells in *C. rotundus* while they were not different in *C. rotundus*. Intercellular spaces between the mesophyll cells were absent in CS while it was present in *C. rotundus*. Below the vascular bundle, a single sclerenchymatous patch was present in the midrib region while in the *C. rotundus*species, it occured in the three different patches (Adams et al.^33^). In powder microscopy of the leaf palisade, cells and spongy parenchymatous cells were seen which is analogous to monocotyledon leaves. In combination with all of this, such characteristics may play an important role into the identification of the plant species.

The Pharmacognostic analysis of rhizomes of *C. rotundus* and CS showed more than 70% similarity^33^. Pharmacognosy is the well-known tool for plant identification, where morphological, organoleptic evaluation, histochemical analysis, and microscopic studies confirmed the authenticity of any plant. Histochemical analysis is a useful tool to detect metabolites of various chemical classes as well as to identify plant secretory structures. Consequently, histochemical evaluation plays a significant role in determining the phytoconstituents as well as type of secretory cells^33^.

There is no HPLC study on CS leaves yet, but there are a few reports, where phenolic compounds have been quantified by using other chromatographic techniques^15,16,19,33^. Usually, the HPLC method below a 40 min run time is considered as the best method for the analysis of plant metabolites. In a few reported methods, these phytochemicals (caffeic acid, catechin, epicatechin, trans-p-coumaric acid, and trans-ferulic acid) were separated up to 40–60 min^34–36^. The important aspect of the new HPLC method developed in the current study was a lower time used for the separation of the phytochemicals (< 30 min). HPLC profiling analysis of *Cyperus rotundus* rhizomes and CS roots showed more than 70% similarity, and CS is recommended as a substituting drug for *C. rotundus*^33^. Flavonoids and phenolic compounds are reported in the leaves of Cyperus species^37^, but no one has quantified these in CS leaves. All of the phenolic compounds quantified in the present study are reported here for the first time. Hydroalcoholic extracts of CS rhizomes showed hypolipidemic activity due to the presence of phenolic compounds that were identified by a qualitative method^38^, but no phenolic compound was not quantified using HPLC or LC–MS analysis. In present study, the CS leaves showed significant phenolic compound content like catechin and epicatechin including other phytochemicals, so these leaves may be used as ingredients in green tea and the decoction of CS leaves used to treat gastric and stomach pain.

There are only a few GC–MS studies on the aroma of CS rhizomes^22^, as the leaves of CS contain same aroma which might be due to the presence of volatile nitrogenous compounds like epi-guaipyridine, guaia-9,11-dienpyridine, and cananodine^22^. The hydrocarbons of CS rhizomes are biogenetically important^39^. All are new phytochemicals reported in the present study. Melezitose is considered as a masking agent in the food industry and a replacement of sugar additives^28^. It is the molecule that suppresses unpleasant taste or sensation, i.e. organoleptic parameters^28^. The presence of a higher content of melezitose in CS makes it sweet by masking the bitter taste. The presence of n-hexadecanoic acid in CS makes it more medicinally potent as n-hexadecanoic acid having anti-inflammatory, anticancer and anti-oxidant properties^29^. The most abundant fatty acids in all tested oat cultivars are linoleic (34.6–38.2%), oleic (30.7–32.2%), and palmitic acid (21.4–22.7%)^30^. The presence of these fatty acids (linoleic, oleic and palmitic acid) in the CS leaf along with melezitose indicates its nutritional value and food importance. Here, the GC–MS/MS study of CS leaf enabled us to identify and report phytochemicals having nutritional value for the first time.

It has been observed that CS leaves are a rich source of K, Ca, Mg, Fe and Zn and all of these are most essential metal ions for proper health function including the treatment of osteoporosis^40^. The Ca rich herbs and food with complimentary elements (like Mg and K) are more nutritional as the absorption of Ca is catalyzed in the presence of Mg and K^41^. Overall, ICP-OES analysis of CS leaves enabled us to quantify the metal ions having the most nutritional significance for the first time.

The reported literature advocates that a majority of the antioxidant activities of plant extracts are derived from the constituent phenolic compounds^42,43^. The antioxidants present in the plants exhibit a wide range of biological activities including antibacterial, antiviral, anti-inflammatory, antiallergic, antithrombotic, and vasodilatory actions. Taking into account the relationship between phenolics and flavonoids and the antioxidant activity of botanicals^8–13^, TPC, TFC and the antioxidant activity (DPPH radical scavenging activity) of the CS extract was evaluated and found significant.

There are limited reports on herbal formulations with antacid potential. In herbal drug development, the dose calculation is the major problem, whereas in the current study, the dose (666 mg or 400 mg etc.) was decided as per the report from Christensen et al.^44,45^. Antacids are the used in gastroesophageal reflux-related symptoms and the FDA recommends them as the first-line treatment for heartburn in pregnancy^46^. It has been reported that cold milk and broccoli have antacid activity comparable with ENO and sodium bicarbonate^47^. The antacid activity of milk and broccoli was correlated with the amount of Ca and antioxidant activity^47^. In the present study, CS leaves are rich in Ca, having antioxidant activity, which may be a reason for its significant antacid activity. Under an artificial stomach model, the combination of CaCO_3_ and *L. aestuans* had significant antacid activity (i.e. > CaCO_3_), and the same trend was observed in the present study. *L. aestuans* is classically defined as an antacid herb in Ghana^45^. The aqueous extract of the fruit rind of Garcinia indica showed antacid activity, but less than CaCO_3_^48^. The methanolic extract of the root of *Tephrosia purpurea* (L.) Pers showed moderate antacid activity^49^. In modern treatment, there are various antacids like Al-based (causes constipation), Mg-based (causes diarrhoea), and NaCO_3_-based (causes Na alteration which causes cardiovascular disease and a high amount of CO_2_), but all have significant risks^45–49^. The antacid activity of CS leaves makes them a more significant traditional medicine as the application of modern drugs themselves create acidity.

The present study depicted the utility of CS leaves as a medicinally and nutrient rich source from agro-waste. This is the first report where phenolic compounds and essential metal ions along with medicinal properties has been reported. Sahini and Mutegoa reported the phenolic, flavonoids, and vitamins in rice agro-waste and its medicinal utility^4^. Sarangi et al. reported the application of agro-waste for the extraction of phenolic compounds having vital medicinal properties^3^. Agro-waste is reported as a source of biogenic nano-materials^5^. Polysaccharides and other phytochemicals rich in agro-waste can be utilized as a new biodegradable and anti-microbial material in packaging materials to enhance the shelf-life of food and meat products^1^.

It has been demonstrated in in-silico studies that CS leaf phytoconstituents have high antioxidant, anticancer, anti-inflammatory, antimicrobial, and antacid potency as compared to standard medications, making them an essential source for novel anti-cancer, antioxidant, antiacid, anti-inflammatory, and anti-microbial medications. In silico studies confirmed the in vitro results obtained in antioxidant, antiacid, and anti-microbial activities. In addition, in silico studies revealed the anti-cancerous and anti-inflammatory potential of CS leaves. In the future, in vivo studies and pharmacological analysis followed by pre-clinical and clinical trials could help to develop CS leaves as a potent antibacterial medicine.

## Conclusion

Overall, this was the first study on CS leaves for the utilization of CS agro-waste as a medicinally nutrient rich source material. The presence of phytochemicals like caffeic acid, catechin, epicatechin, trans-p-coumaric acid, and trans-ferulic acid along with fatty acids and essential metal ions makes it nutritional rich. The TPC, TFC and antioxidant assays reflected the medicinal potential and health benefits of CS leaves. The presence of micronutrients (such as Ca, Na, K, Mg, Fe, Zn, etc.) and fatty acids (such as linoleic, oleic, palmitic acid, etc.) along with phenolic compounds (such as catechin, epicatechin, etc.) in CS leaves highlighted its high nutritional value. The methanolic extract (100 mg/mL) of CS leaves showed for the first time zone of inhibition trends as: *Staphylococcus aureus* (15 ± 2 mm) > *Escherichia coli* (12 ± 2 mm) > *Pseudomonas aeruginosa* (8 ± 2 mm) > *Salmonella typhi* (8 ± 2 mm). In silico studies confirmed antioxidant, antiacid, and anti-microbial in vitro results. In addition, in silico studies revealed the anti-cancerous and anti-inflammatory potential of CS leaves, which needs further validation. Undoubtedly, this study showed the medicinal significance of the under-utilized part of CS and conversion of agro-waste into mankind activity as a pharmaceutical potent material. In the future, there is a need to perform toxicological and pharmacological studies of CS leaves before it is to be used as a potential drug.

## Materials and methods

### Plant material

The leaves of CS or *Cyperus scariosus* R.Br. (Nagarmotha) were collected from local fields of the Central Ayurveda Research Institute, Jhansi, in May, 2023. The plant was authenticated by the Dr. Jagdish C. Arya and Dr. Shyam Baboo Prasad with the help of a digital herbarium (https://powo.science.kew.org/taxon/305856-1). A voucher specimen of the plant was preserved in the herbarium section of the Institute (Herbarium specification No is 1011). Experimental research and field studies on plants (either cultivated or wild), including the collection of plant material, must comply with relevant institutional, national, and international guidelines and legislation.

### Chemicals and reagents

HPLC grade water, acetonitrile, methanol, formic acid, DPPH, trolox, sodium carbonate, aluminum chloride, sodium hydroxide, sodium nitrite, elemental standards (As, Cd, Hg, Pb, Na, K, Ca, Mg, Mn, Zn, Al, Fe, and Cu), nitric acid pepsin, sodium chloride, hydrochloric acid, and calcium carbonate were obtained from Merck KGaA, Darmstadt, Germany. Gallic acid, quercetin, catechin, epicatechin, trans-p-coumaric acid, trans-ferulic acid, and caffeic acid were procured from TCI Chemicals, Japan. The Folin–Ciocalteu reagent and tris–HCl buffer (pH 7.4) were obtained from Himedia Laboratories Pvt. Ltd, India. Analytical grade phloroglucinol, safranin, Fast green, dragendroff’s solution, sulphuric acid, hydrochloric acid, sodium hydroxide, sudan red III, ferric chloride, acetic acid, and an iodine solution were obtained from LOBA Chemie.

Test strains including gram-negative pathogens (*Escherichia coli* (MTCC No. 1885), *Pseudomonas aeruginosa* (MTCC No. 424) and *Salmonella typhi* (MTCC No. 733)) and a gram-positive pathogen (*Staphylococcus aureus* (MTCC No. 1430^T^) were procured from MTCC Chandigarh, India. Culture media including Nutrient Broth (NB), Nutrient Agar (NA) and Mueller Hinton Agar (MHA) were purchased from Himedia Laboratories Private Limited, Thane, Maharashtra, India. Petri dishes (90 mm), cork borer (6 mm), cotton swabs and the McFarland standard were also procured from Himedia.

### Method of pharmacognostic evaluation

#### Macroscopic and organoleptic analysis

Macroscopic characterstics and organoleptic characteristics of the CS leaves were observed and noted^23^.

#### Microscopic characterization

Freehand sections (T.S.) of a leaf were taken. The obtained thin section was stained with safranin (1% safranin solution in 50% ethyl alcohol). The T.S. image was taken using an Olympus BX 43 and LC 30 camera^23^.

#### Powder microscopy

A powder sample (1 mg) was stained with an iodine solution (composed of 2 g iodine and 3 g potassium iodide in 100 mL of water) and then was mounted in 50% glycerine on one slide. On another slide, a sample of 1 mg was stained with phloroglucinol (0.1% w/v) and diluted with HCl (10% solution) then washed in water and after that mounted in 50% glycerine. Moreover, on another slide, a 1 mg sample was stained with safranin (1% safranin solution in 50% ethyl alcohol). The microscopical characteristics of the prepared slides were observed using an Olympus BX 43 and LC 30 camera^23^.

#### Quantitative microscopy and histochemistry

Quantitative microscopic analysis was performed to define the stomatal number, stomatal index, vein islet number, vein termination number and palisade ratio of leaves. Histochemical evaluation of the leaves was conducted (Kumar et al.^9^).

### Sample extraction for HPLC, GC–MS/MS analysis

5 g of the powdered sample was mixed with 50 mL of methanol and was sonicated for 15 min. The mixture was then centrifuged at 5000 rpm for 5 min and the supernatant was filtered using a 0.22 μm syringe filter. The solution, thus, obtained was used as the sample solution for HPLC, GC–MS/MS analysis and for antioxidant activity evaluation.

### HPLC instrumentation and chromatographic conditions

HPLC analysis was carried out by an Agilent 1260 Infinity II LC system (Agilent Technologies, USA), equipped with an autosampler, 100 μL syringe, flexible pump, multicolumn thermostat (MCT), and diode array detector (DAD) with a wavelength range 200–800 nm and the data analysis was carried out using OpenLab CDS ChemStation Edition software (OpenLab Chemstation C.01.10; https://www.agilent.com/en/product/software-informatics/analytical-software-suite/chromatography-data-systems/openlab-chemstation). Chromatographic conditions were as follows: mobile phase A: water acidified with 0.1% formic acid at pH 3 and B: acetonitrile. The gradient program was 0.01–5 min 90% A and 10% B, 5.01–10 min 88% A and 12% B, 10.01–15 min 86% A and 14% B, 15.01–21 min 84% A and 16% B, 21.01–25 min 80% A and 20% B, 25.01–32 min 78% A and 22% B, and 32.01–40 min 70% A and 30% B. A solvent flow rate of 0.8 mL/min and temperature of 30 °C were used. The calibration curves were produced in the range of 1–60 ng/µL. The experiments were performed in triplicate. The targeted phytochemicals in CS were quantified at different wavelengths, i.e., catechin and epicatechin at 280 nm, and caffeic acid, trans-p-coumaric acid, and trans-ferulic acid at 326 nm.

### HPLC method validation

The developed HPLC method was also validated as per ICH guidelines^50,51^. Specificity was evaluated by comparing the absorption spectra of the standards with the corresponding peaks in the sample and by injecting a sample blank and standard solution. Linearity was demonstrated by injecting standard mixture solutions at 1–60 ng/μL for caffeic acid, trans-p-coumaric acid, and trans-ferulic acid and 5–60 ng/μL for catechin and epicatechin (M1: 1 ng/µL; M2: 2 ng/µL; M3: 5 ng/µL; M4: 10 ng/µL; M5: 20 ng/µL; M6: 40 ng/µL; M7: 60 ng/µL). The accuracy of the method was assessed by performing analysis on three spiked samples having known concentrations of targeted phytochemicals and calculating the percent recovery. Precision was evaluated by injecting three concentrations of the standards on the same day (intra-day) and on three different days (inter-day). The LOD and LOQ were evaluated by using the slope (s) and standard deviation of the intercept (σ) of the calibration curve by the formulae LOD = 3.3σ/s and LOQ = 10σ/s. Robustness was assessed under variable chromatographic conditions including changes in the mobile phase composition, flow rate, column temperature, etc.

### GC–MS/MS instrumentation and chromatographic conditions

Gas chromatography–mass spectrometry (GC–MS/MS) analysis of CS was carried out by employing an Agilent 8890 GC System in combination with 7000D GC/TQ by using two combined Agilent HP-5MS UI columns ((5%-phenyl)-methylpolysiloxane nonpolar, 15 m × 250 μm × 0.25 μm) following the procedure of our recent studies^23^. The separation of phytochemicals present in methanolic extract was done by employing helium as a carrier gas at constant flow rates of 1.0 mL/min and 1.1 mL/min in Columns I and II, respectively. The extract solution (10 mg/mL) with an injection volume of 2 μL and split ratio of 10:1 was injected into the column by an autosampler. During the chromatographic run, the initial oven temperature was 60 °C, which further increased to 100 °C at a rate of 4 °C/min (hold time 5 min); which further increased to 160 °C at a rate of 15 °C/min (hold time 2 min); which further increased to 200 °C at a rate of 5 °C/min (hold time 15 min). The conditions of the mass detector were: source temperature at 230 °C, mass detector (MSD) transfer line temperature at 280 °C, ionization mode electron impact (EI) at 70 eV scan time 300 ms (0.3 s), in positive and MS2 scan mode with a solvent delay of 3 min and a mass range of 50–650. The pressure of the collision gas N_2_ and quench gas He were set at 1.5 mL/min and 2.25 mL/min, respectively. The mass spectra of the major peaks, i.e., major phytochemicals obtained in GC–MS chromatograms, were compared with the database stored in the GC–MS NIST library.

### Elemental analysis

The 0.5 g of the powdered sample was digested with 10 mL of nitric acid using an Anton Paar Multiwave GO Plus Microwave Digestion System. The digestion program included a ramp time of 25 min at 190 °C and then hold that time for 20 min. The volume of the resultant solution was made up to 25 mL with deionized water and the solutions were filtered to remove any insoluble impurities. The solutions were used as such for heavy metal analysis and were diluted with 2% nitric acid for the essential elemental analysis. The elemental analysis was carried out using an Agilent 5800 ICP-OES system equipped with a SPS 4 Autosampler. The elemental analysis on ICP-OES was done as per the procedure mentioned in a recent study^13^ and the detailed ICP-OES conditions are mentioned in the Supplementary data (Table S1).

### Total phenolic content and total flavonoid content

Total phenolic content (TPC) was evaluated by the Folin–Ciocalteu colorimetric assay, as described in the AOAC methods, using a solution of gallic acid as a standard phenol solution (10–1000 mg/L)^52^. In brief, 50 µL (of a 10 mg/mL sample extract solution) was taken in a test tube, 3950 µL of distilled water and 250 µL of a Folin–Ciocalteu reagent were added and 750 µL of an aqueous sodium carbonate solution (20%) was added after 6 min. The resultant solutions were incubated at 30 °C for 2 h. The absorbance of the resultant solutions was measured at 765 nm against a blank. The quantitative measurements were performed on the basis of a standard calibration curve of eight points of aqueous gallic acid solutions (10, 25, 50, 100, 250, 500, 750, and 1000 mg/L). TPC was expressed as gallic acid equivalents (GAE) in milligrams per gram of the powdered sample. All of the measurements were carried out in triplicate.

Total flavonoid content (TFC) was evaluated by the aluminum chloride colorimetric assay, as described in the AOAC method, using a solution of quercetin as a standard flavonoid solution (50–2000 mg/L)^53^. In brief, 400 µL (of 10 mg/mL sample extract solutions) was taken in a test tube, 300 µL of an aqueous sodium nitrite solution (5%) was added and 300 µL of an aqueous aluminum chloride solution (10%) was added after 5 min and then 2 mL of a 1 M sodium hydroxide solution was added after 6 min. To the resulting solutions, 2.4 mL of distilled water was added and the solutions were incubated at 30 °C for 2 h. The absorbance of the resulting solutions was measured at 510 nm against a blank. Quantitative measurements were performed on the basis of a standard calibration curve of nine points of methanolic quercetin solutions (50, 100, 250, 500, 750, 1000, 1500, 2000, and 3000 mg/L). TFC was expressed as quercetin equivalents (QCE) in milligrams per gram of the powdered sample. All of the measurements were carried out in triplicate. The TPC and TFC assays were carried out using a Labindia UV3200 UV–Vis spectrophotometer.

### Antioxidant activity

Antioxidant activity was estimated by the DPPH assay, as per the procedure described by Gandhi et al.^10^, by using trolox as a standard antioxidant^42^. In brief, to 2 mL of a 0.1 mM methanolic DPPH solution, 1.6 mL of a 0.1 M Tris–HCl buffer (pH 7.4) and 400 µL of the sample was added in a test tube. The resulting solutions were kept under dark for 30 min at room temperature and the absorbance of the solutions was recorded at 517 nm against a blank. A solution containing 400 µL of methanol instead of the sample was used as the control. The below-mentioned equation was used for the calculation of inhibition ratio (%):

$$Inhibition \;ratio \left(\%\right)= \frac{{A}_{c}-{A}_{s}}{{A}_{c}} \times 100$$

where A_c_ and A_s_ are the absorbance of control and sample, respectively.

The plot of inhibition ratio against the concentration of the sample was not a straight line. The two points enclosing the inhibition ratio of 50% were joined by a regression line (y = mx + c) and the IC_50_ value was calculated by substituting y with 50. The IC_50_ values of the sample solutions and trolox were estimated and the DPPH radical scavenging activity of the extract was expressed in terms of trolox equivalent antioxidant activity (TEAC). The below-mentioned expression was used for calculating TEAC:

$$TEAC= \frac{{IC}_{50} \, of \, Trolox \, (mg/mL)}{{IC}_{50} \, of \, sample \, (mg/mL)}$$

#### Antacid activity

The dried 666 mg of the powdered CS leaf sample, 400 mg of CaCO_3_, 200 mg of CaCO_3_, gelusil (an antacid drug), and the combination of CS leaves and CaCO_3_ were added to a 250 mL beaker and 90 mL of tap water was added with the mixture warmed to 37 °C and stirred constantly at 30 rpm by using a temperature controlled magnetic stirrer. The resulting test solutions were titrated by using the prepared artificial gastric acid (800 U/L pepsin in 34 mM NaCl having a pH 1.2 adjusted by using HCl) to the end point of pH 3 as per Fordtran’s model^44,45^. The rate of secretion of the artificial gastric acid was kept at 3 mL/min and pH was recorded at a regular time interval of 1 min.

#### Antimicrobial analysis

Culture media was weighed and autoclaved at 121 °C and at 15 psi pressure for 20 min. Around 20–40 mL of the autoclaved media was poured into 90 mm Petri dishes in aseptic conditions under laminar airflow and were kept overnight to solidify. The bacterial cultures of gram-positive and gram-negative bacteria were maintained on nutrient agar medium for 24 h at 37 °C^54^. Fresh inoculums of the test microorganism in nutrient broth were prepared from freshly grown cultures on a nutrient agar plate. Each strain was adjusted at a concentration of 10^8^ cells/mL using the 0.5 McFarland standard and 10 µL of inoculum of each test microorganism was spread on MHA agar plates, respectively. The agar well diffusion method was used to evaluate the antibacterial activity of the methanolic extract of *Cyperus scariosus* (C.S) leaves (conc. 100 mg/mL). The MHA agar plates were spread with 10 µL of the test microorganism which were then punched with a sterile cork borer (diameter of 6 mm) for well preparation. A volume of 200 µL each of the test extract, a standard antibiotic Kanamycin (1 mg/mL) as a positive control, and solvent (methanol) as a negative control were introduced into respective wells on each MHA agar plate and the plates were incubated at 37 °C for 24 h^54^.

### Computational studies

#### Molecular docking study

In silico analysis of the selected phytochemicals (confirmed using HPLC and GC–MS) of CS leaves were performed using Schrodinger suite v 13.1 Maestro. The preparation of ligand and protein settings were explored as per the procedure mentioned by Sastry et al.^55^, including the targets, PDB codes, and the number of active ligands for the exploratory set. In silico analysis was performed for the antacid (PDB Id: 2VCZ), antioxidant (PDB Id: 1G1T), anticancer (PDB Id: 6BL4), antimicrobial (PDB Id: 1OG5), and anti-inflammatory (PDB Id: 5X14) activities against protein receptors^55^. For molecular modelling, the usual protein structure file retrieved from the PDB is inappropriate, and water molecules, metal ions, cofactors, and co-crystallized ligands are all present in typical PDB structure files. Initially, the protein was preprocessed, optimized, and decreased (to minimum energy) before the protein preparation wizard was made^55^. The final result was a fine, hydrogenated ligand and ligand–receptor complex structure, which was then used to analyze various Schrodinger modules for in silico screening^55^.

#### Ligand preparation and grid generation

In order to get the best possible docking results, the ligands were prepared using the Maestro v 13.1 Lig-Prep modules^56–58^. The ligand structures in the protein–ligand complex must be faithfully portrayed by the docked structures. Therefore, the ligand structure must meet the Glide docking program requirements, and three dimensions ought to be necessary^56–58^. The only geometric characteristics that Glid alters are the ligands and torsional coordinates; as a result, these must be altered beforehand. Each must only contain a single molecule, free of any counterions, solvent molecules, or covalent receptor attachments. Their valences must include hydrogen and be properly protonated with physiological pH levels of around 7^56–58^. The grid was created by the receptor grid creation module in Maestro version 13.1. A grid surrounds the binding site of the co-crystallized ligand, allowing other molecules to bind there while keeping the co-crystallized ligand out^59^.

### Network pharmacology

The integrated network pharmacology approach was adopted to evaluate bioactive phytochemicals of CS observed in HPLC and GC–MS experiments as reported by the Harakeh et al.^60^. Moreover, STITCH database was also used along with swisstarget prediction webserver.

### Statistical analysis

All of the studies were performed in triplicate and t-tests were applied by using Origin 2019 Software. All the results were reported as mean ± SD.

### Permissions and identification of plant material

Permissions were obtained to collect the plant material. Moreover, this is common weed and treated as agro-waste, so no specific permission required. The plant material was collected and authenticated by the Dr. Shyam Baboo Prasad. The herbarium was submitted in Institute (a public herbarium) with accession number No is 1011.

### Ethical approval

The authors declare that this study required no ethical approval, as it is an observational study.

### Supplementary Information

Supplementary Information.

## Data Availability

The datasets generated and/or analyzed during the current study are not publicly available but are available from the corresponding author on reasonable request. All additional data mandatory for this article is provided in a supplementary file.

## Abbreviations

CS: *Cyperus scariosus*
TPC: Total phenolic content
TFC: Total flavonoid content
DPPH: 2,2 Diphenyl 1 picrylhydrazyl
TEAC: Trolox equivalent capacity
GAE/g: Gallic acid equivalent per gram
QCE/g: Quercetin equivalent per gram
HPLC: High performance liquid chromatography
LC–MS: Liquid chromatography–mass spectrometry
GC–MS: Gas chromatography–mass spectrometry
API: Ayurvedic Pharmacopeia of India
AFI: Ayurvedic Formulary of India
DAD: Diode array detector
