# Supplementary material for: Chemoprofiling and medicinal potential of underutilized leaves of Cyperus scariosus
Source: Sci Rep. 2024 Mar 27;14:7263. doi: 10.1038/s41598-024-58041-7 (PMC10973434; doi:10.1038/s41598-024-58041-7)
Supplement: Supplementary file 1 — Supplementary Information. [file 41598_2024_58041_MOESM1_ESM.pdf]

SUPPLEMENTARY DATA

Exploration of neglected and under-utilized leaf part of *Cyperus scariosus* as a medicinally and nutrient rich source from agro-waste

Yashika Gandhi[1#], Vijay Kumar[1#*], Gagandeep Singh[1#], Shyam Baboo Prasad[1#], Sujeet K. Mishra[1#], Hemant Soni[1#], Hemant Rawat[1#], Simranjeet Singh[2#*], Vaibhav Charde[1], Akhil Gupta[1], Daljeet Singh Dhanjal[3], Sudhanshu Kumar Jha[1], Smriti Tandon[1], Prateeksha Bhagwat[1], Jagdish C. Arya[1], Praveen C. Ramamurthy[2], Rabinarayan Acharya[4], and Thomas J. Webster[5]

1. Central Ayurveda Research Institute, Jhansi, Uttar Pradesh – 284003, India.
2. Indian Institute of Sciences, Bangalore – 560012, India.
3. Lovely Professional University, Phagwara, Punjab – 144411, India.
4. Central Council for Research in Ayurvedic Sciences, New Delhi – 110058, India.
5. School of Health Sciences and Biomedical Engineering, Hebei University of Technology, Tianjin, China; School of Engineering, Saveetha University, Chennai, India, and Program in Materials Science, UFPI, Teresina, Brazil.

Taxonomic description

Cyperus scariosus is a glabrous herb with soft angular stem. Stolons are slender, clothed by elliptic, acute, lax striate concolorous scales, stem 40-120 cm long, triquetrous at top leaves are variable, usually short (less than 1/3 stem), narrow, weak, and umbels are slender, contracted, rays slender sometimes up to 3 inch long, and bracts are nearly always as the leaves i.e. hardly any when leaves short, exceeding inflorescence when leaves longish, and spikelets are linear pale straw-colour and rhizome are very short, woody, stolons, lateral shoots from base of stem immediately ascending, glumes scarcely imbricate in fruit. The rhizome of this plant contains an amber or light brown viscous essential oil.

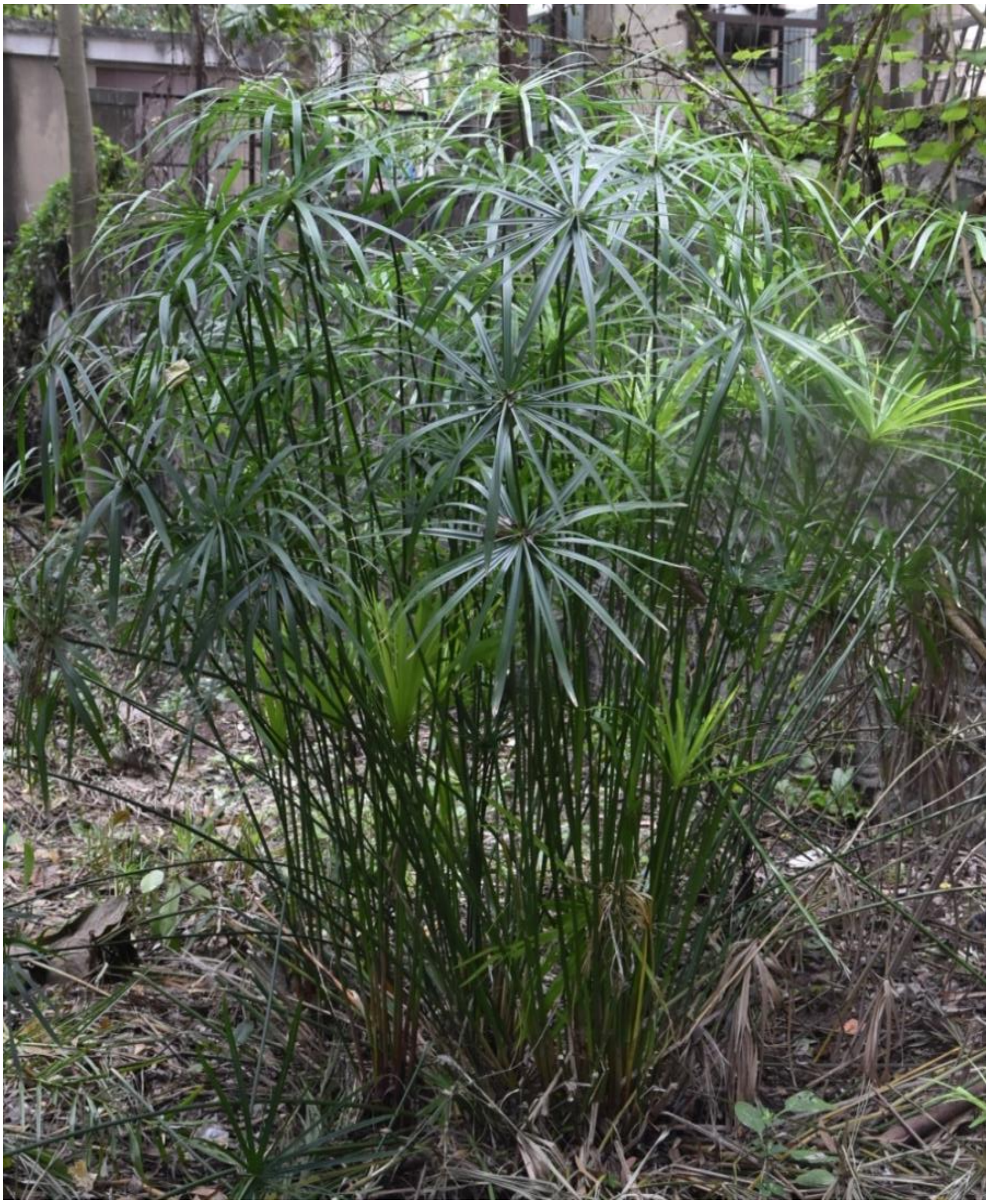

Whole plant of *Cyperus scariosus* R.Br.

Distribution

Cyperus scariosus R.Br. occurs in Myanmar, Bangladesh, India, Papua New Guinea, Australia etc. (Xue et. al., 2023). In India, Distribution map of plant is available in public domain. (India Biodiversity portal). Cyperus scariosus R.Br. (Nagarmotha) belongs to the family Cyperaceae. It is a perennial plant (Prasad VP, Simpson DA 2012; Kumar A, Chahal KK 2017). This plant is found in and around the rivers, and waterfalls, especially found in damp or marshy regions as well as along the coastal areas (Kumar A, Chahal KK 2017; Srivastava RK, Singh A 2014). Nagarmotha is widely distributed in the eastern and southern parts of India (CP Khare). Also, found in Bihar, Orissa, Chhattisgarh, in damp places in Madhya Pradesh, Uttar Pradesh, and Bengal. [2 Kumar Cyperus scariosus R.Br. (Nagarmotha) belongs to the family Cyperaceae. It is a perennial plant (Prasad VP, Simpson DA 2012; Kumar A, Chahal KK 2017). Nagarmotha is found in and around the rivers, and waterfalls, especially found in damp or marshy regions as well as along the coastal areas (Kumar A, Chahal KK 2017; Srivastava RK, Singh A 2014). Nagarmotha is widely distributed in the eastern and southern parts of India (CP Khare). Also, found in Bihar, Orissa, Chhattisgarh, in damp places in Madhya Pradesh, Uttar Pradesh, and Bengal (Kumar A, Chahal KK 2017). Moreover, also grows in Australia, Malesia, China, South Africa, and Pacific Islands (Prasad VP, Simpson DA 2012; Kumar A, Chahal KK 2017] A, Chahal KK 2017). Moreover, also grows in Australia, Malesia, China, South Africa, and Pacific Islands (Prasad VP, Simpson DA 2012; Kumar A, Chahal).

References:

1. Prasad VP, Simpson DA. The status of Cyperus pertenuis and Cyperus scariosus (Cyperaceae). Kew Bulletin. 2012 Mar;67(1):93-6.
2. Kumar A, Chahal KK, Kataria D. A review on phytochemistry and pharmacological activities of Cyperus scariosus. Journal of Pharmacognosy and Phytochemistry. 2017;6(1):510-7.
3. Srivastava RK, Singh A, Srivastava GP, Lehri A, Niranjan A, Tewari SK, Kumari K, Kumari S. Chemical constituents and biological activities of promising aromatic plant nagarmotha (Cyperus scariosus R. Br.): A review. InProc Indian Natn Sci Acad 2014 Sep (Vol. 80, No. 3, pp. 525-536).
4. CP Khare. Indian Medicinal Plant, An illustrated dictionary, Janak Puri, New Delhi. Pg. 195
5. India Biodiversity Portal (https://indiabiodiversity.org/species/show/229437)
6. India Biodiversity Portal (https://indiabiodiversity.org/observation/show/1752667?lang=en)
7. Worldwide map: https://bie.ala.org.au/species/https://id.biodiversity.org.au/node/apni/2891944
8. Xue, BX., He, RS., Lai, JX. et al. Phytochemistry, data mining, pharmacology, toxicology and the analytical methods of Cyperus rotundus L. (Cyperaceae): a comprehensive review. Phytochem Rev 22, 1353–1398 (2023). https://doi.org/10.1007/s11101-023-09870-3

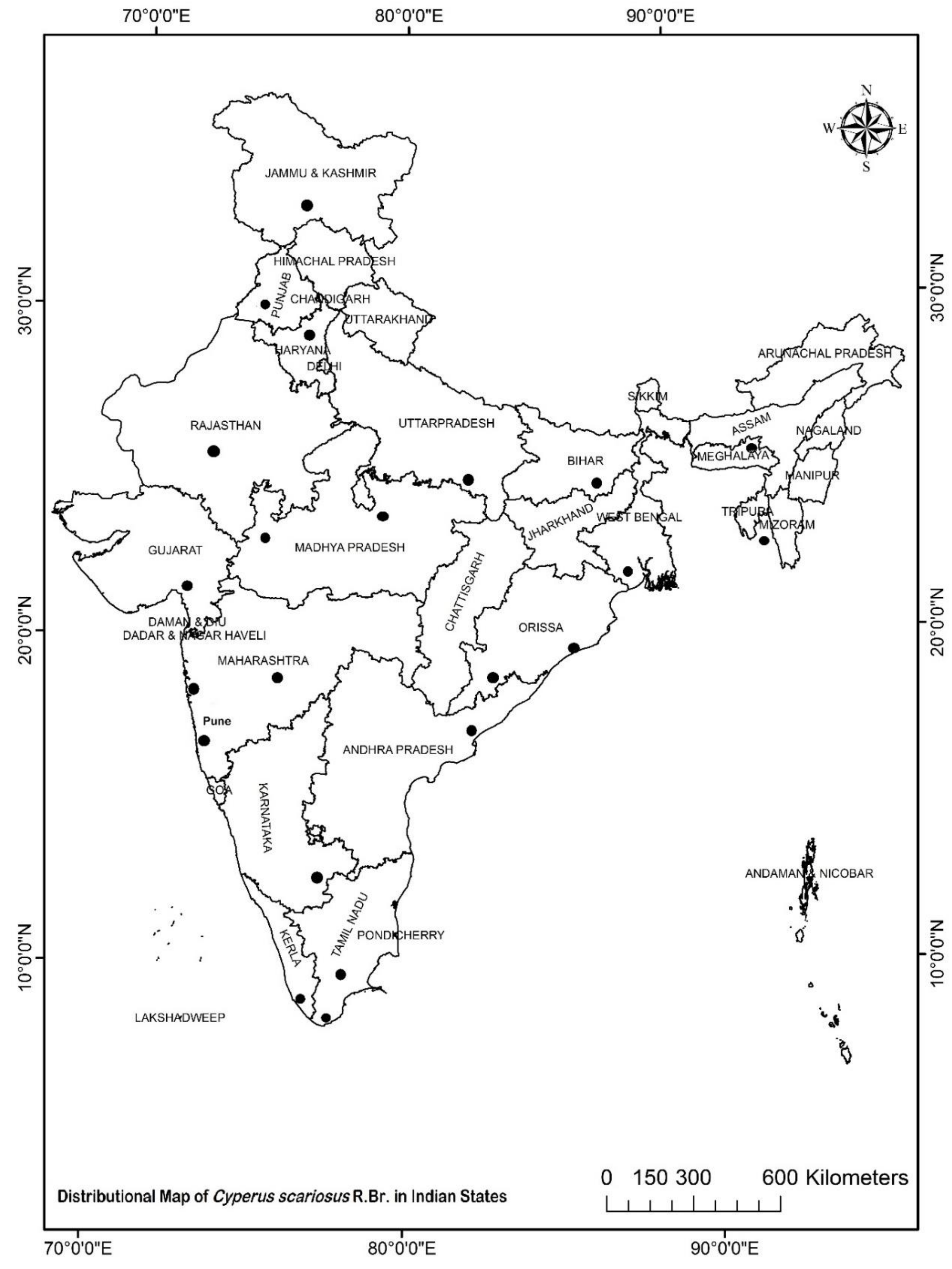

Distribution in India.

Reference: India Biodiversity Portal (https://indiabiodiversity.org/species/show/229437)

(https://indiabiodiversity.org/observation/show/1752667?lang=en)

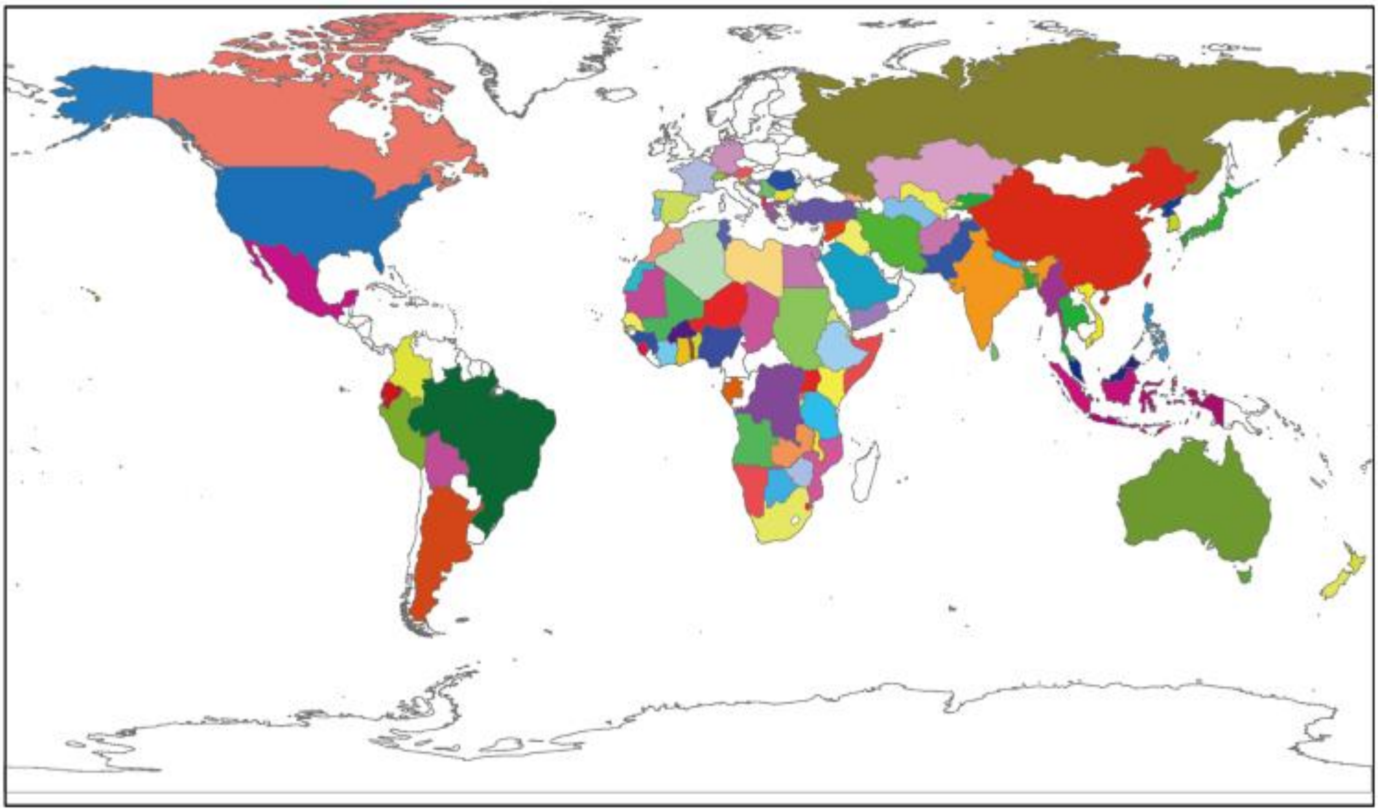

Worldwide distribution (Xue et. al., 2023)

Invasive problems and eradication

Cyperus scariosus R.Br. and *Cyperus rotundus* are one of the most invasive weeds known, having spread out to a worldwide distribution in tropical and temperate regions. Its existence in a field significantly reduces crop yield, both because it is a tough competitor for ground resources, and because it is allelopathic, the roots releasing substances harmful to other plants. Similarly, it also has a bad effect on ornamental gardening. The difficulty to control it is a result of its intensive system of underground tubers, and its resistance to most herbicides. It is also one of the few weeds that cannot be stopped with plastic mulch.Weed pulling in gardens usually results in breakage of roots, leaving tubers in the ground from which new plants emerge quickly. Ploughing distributes the tubers in the field, worsening the infestation; even if the plough cuts up the tubers to pieces, new plants can still grow from them. In addition, the tubers can survive harsh conditions, further contributing to the difficulty to eradicate the plant. Hoeing in traditional agriculture of South East Asia does not remove the plant but leads to rapid regrowth.

Most herbicides may kill the plant's leaves, but most have no effect on the root system and the tubers. Glyphosate will kill some of the tubers (along with most other plants) and repeated application can be successful. Halosulfuron-methyl will control nut grass after repeated applications without damaging lawns. The plant does not tolerate shading and 2,4-dichlorophenoxyacetic acid (2,4-D) slows its growth in pastures and mulch crops.

Table S1. ICP-OES Conditions for elemental analysis.

Common Conditions			
**Replicate Count**	3	**Read Time (s)**	5
**Pump Speed (rpm)**	12	**RF Power (KW)**	1.2
**AVS67 Enabled True**	True	**Stabilization Time (s)**	5/ 15
**Sample Uptake Time (s)**	25	**Viewing Mode**	Axial/ Radial
**Uptake Delay (s)**	14.2	**Viewing Height (mm)**	8
**Sample Uptake Fast Pump**	True	**Nebulizer Flow (L/Min)**	0.7
**Uptake Rate (mL/min)**	27.8	**Plasma Flow (L/min)**	12
**Injection Rate (mL/min)**	9.6	**Aux Flow (L/min**	1
**Bubble Injection Time (s)**	1.799999 9999999998	**Makeup Flow (L/min)**	0
**Preemptive Rinse Time (s)**	2.8	**Oxygen Percent (%)**	0

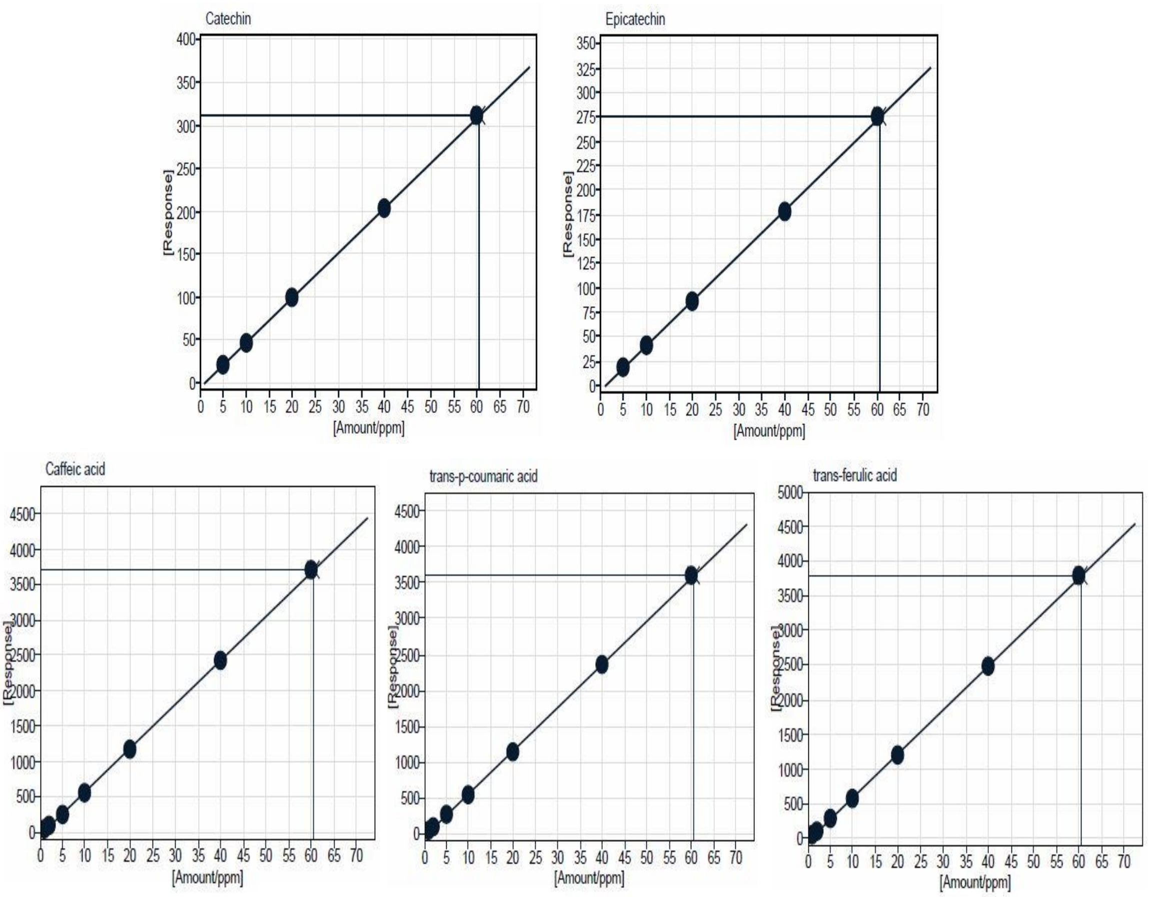

Figure S1. HPLC derived calibration curves of analysed phytochemicals.

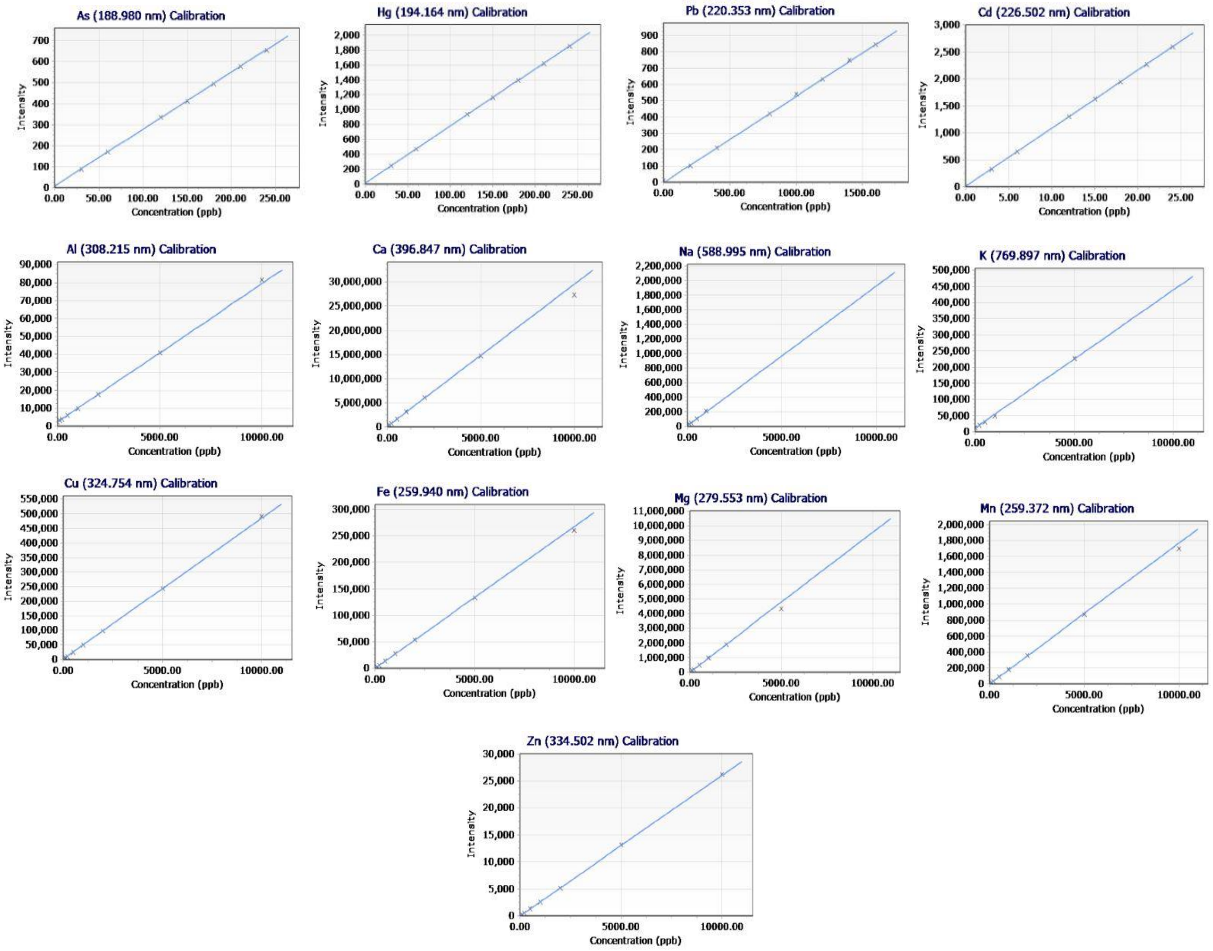

Figure S2. ICP-OES derived calibration curves of different elements.

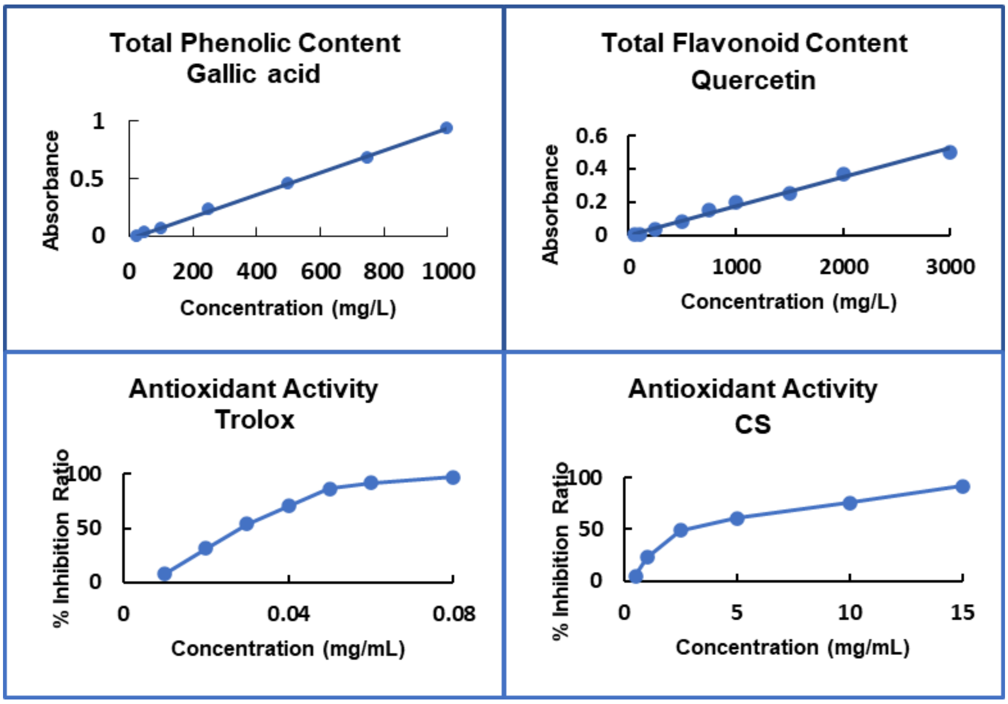

Figure S3. Calibration curves of Gallic acid and Quercetin and antioxidant activity of Trolox and CS extract.

Figure S4: Chemical structure composition of phytoconstituents along with standard drug under in-silico analysis.

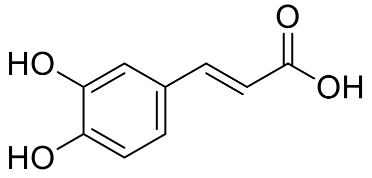<br>Caffeic acid<br>Chemical structure of Caffeic acid **($C_9H_8O_4$)**	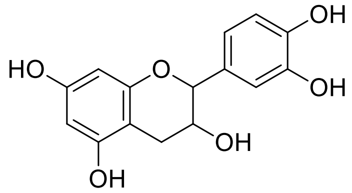<br>epicatechin<br>Chemical structure of Epicatechin **($C_{15}H_{14}$O6)**
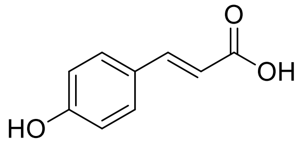<br>trans-p-coumaric acid<br>Chemical structure of trans-p-coumaric acid **($C_9H_8O_3$)**	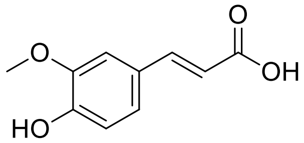<br>trans-ferulic acid<br>Chemical structure of trans-ferulic acid **($C_{10}H_{10}O$)**
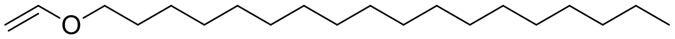<br>Stearyl vinyl ether<br>Chemical structure of Stearyl vinyl ether **($C_{20}H_{40}O$)**	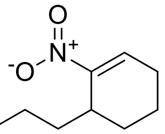<br>6-Butyl-1-nitro-1-cyclohexene<br>Chemical structure of 6-Butyl-1-nitro-1-cyclohexene **($C_{10}H_{17}NO$)**
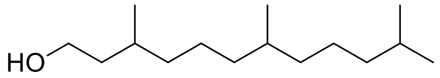<br>3,7,11-Trimethyl-1-dodecanol<br>Chemical structure of 3,7,11-Trimethyl-1-dodecanol **($C_{15}H_{32}O$)**	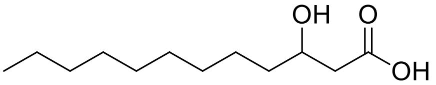<br>beta-Hydroxydodecanoic acid<br>Chemical structure of β-Hydroxydodecanoic acid **($C_{12}H_{24}O_3$)**
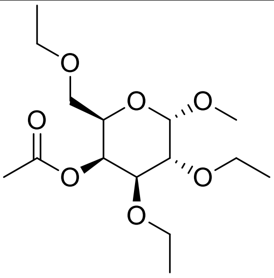<br>Methyl 4-O-acetyl-2,3,6-tri-O-ethyl-alpha-d-galactopyranoside<br>Methyl 4-O-acetyl-2,3,6-tri-O-ethyl-α-d-galactopyranoside **($C_{15}H_{28}O$)**	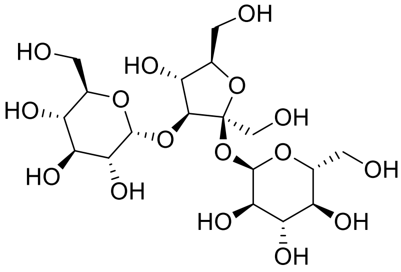<br>Melezitose<br>Chemical structure of **Melezitose ($C_{18}H_{32}O_{16}$)**
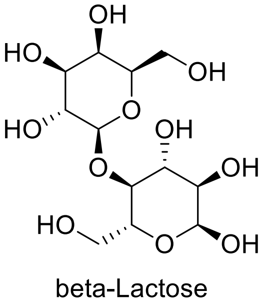<br>beta-Lactose<br>Chemical structure of β-lactose **($C_{12}H_{22}O_{11}$)**	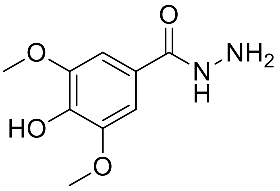<br>4-Hydroxy-3,5-dimethoxybenzohydrazide<br>Chemical structure of 4-Hydroxy-3,5-dimethoxybenzohydrazide **($C_9H_{12}N_2O_4$)**

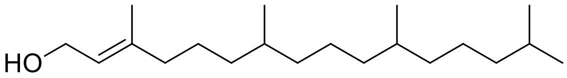 3,7,11,15-Tetramethyl-2-hexadecen-1-ol, Chemical structure of 3,7,11,15-Tetramethyl-2-hexadecen-1-ol, **($C_{20}H_{40}O$)**	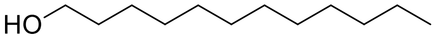 1-Dodecanol Chemical structure of 1-Dodecanol **($C_{12}H_{26}O$)**
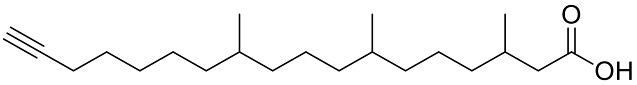 3,7,11-trimethyl-17-Octadecynoic acid Chemical structure of 3,7,11-trimethyl-17-Octadecynoic acid **($C_{21}H_{38}O_2$)**	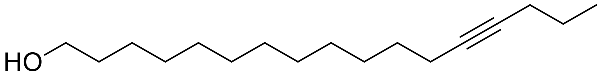 13-Heptadecyn-1-ol Chemical structure of 13-Heptadecyn-1-ol **($C_{17}H_{32}O$)**
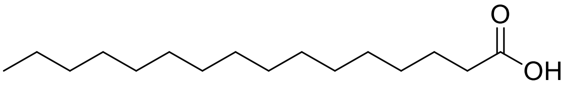 Palmitic acid Chemical structure of Palmitic acid **($C_{16}H_{32}O_2$)**	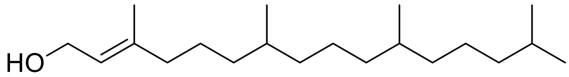 Phytol Chemical structure of Phytol **($C_{20}H_{40}O$)**
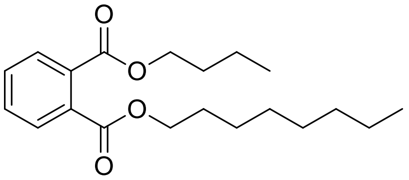 Butyl octyl phthalate Chemical structure of Butyl octyl phthalate **($C_{20}H_{30}O_4$)**	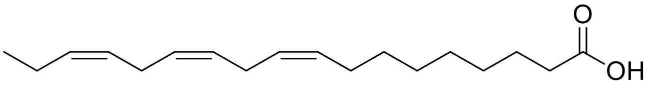 Linolenic acid Chemical structure of Linolenic acid **($C_{18}H_{30}O_2$)**
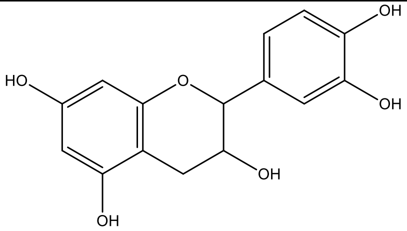 catechin Chemical structure of Catechin **($C_{15}H_{14}O_6$)**	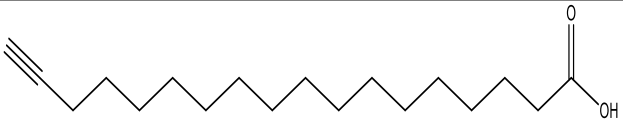 17-octadecynoic acid Chemical structure of 17-octadecynoic acid **($C_{18}H_{32}O_2$)**
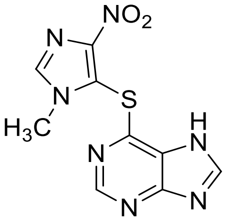 Chemical structure of Azathioprine **($C_9H_7N_7O_2S$)**	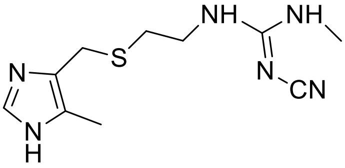 Chemical structure of Cimetidine **($C_{10}H_{16}N_6S$)**
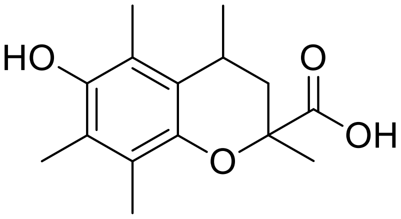 Chemical structure of Trolox **($C_{14}H_{18}O_4$)**	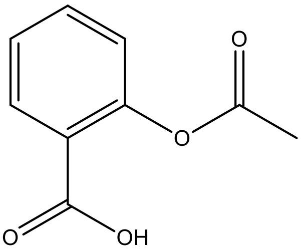 Chemical structure of **Aspirin ($C_9H_8O_4$)**

Figure S5:

PDB ID: **2VCZ**: Complex structure of prostaglandin D2 synthase at 1.95A

Classification: ISOMERASE

Organism(s): Homo sapiens

Expression system: Escherichia coli BL21(DE3)

Mutation(s): No

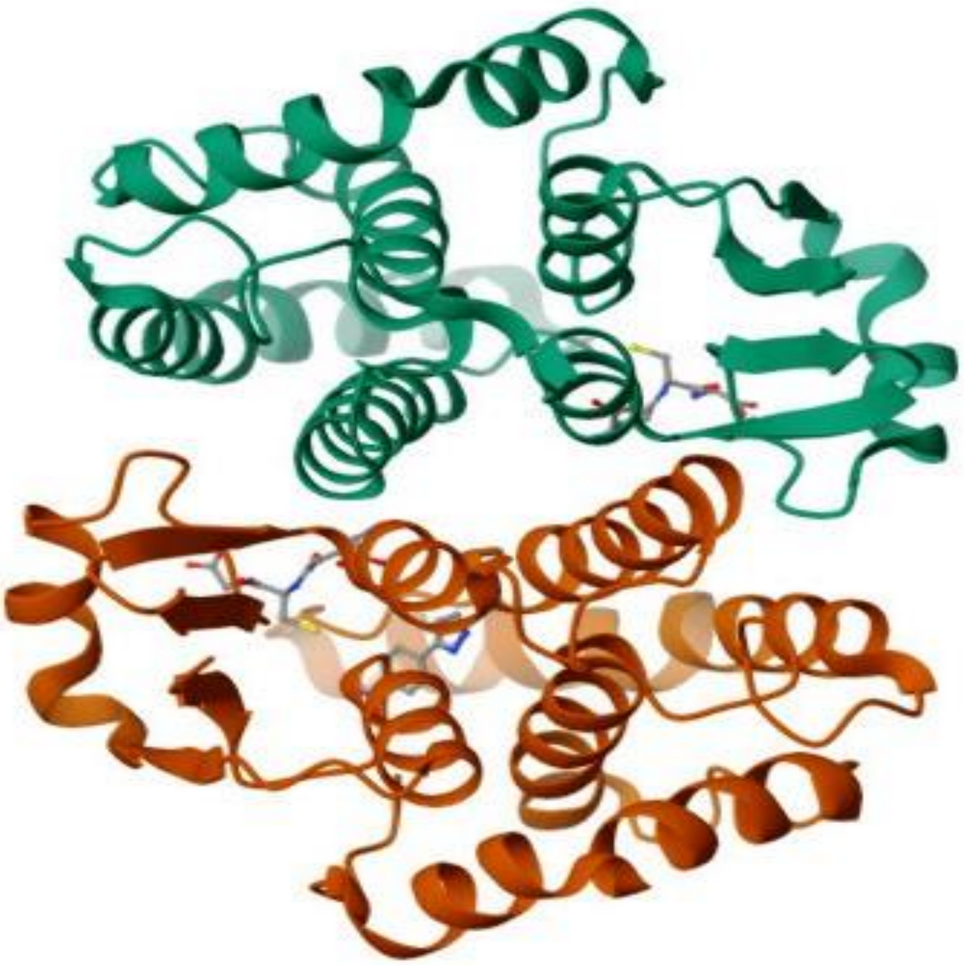

3D- Structure of protein (2VCZ)

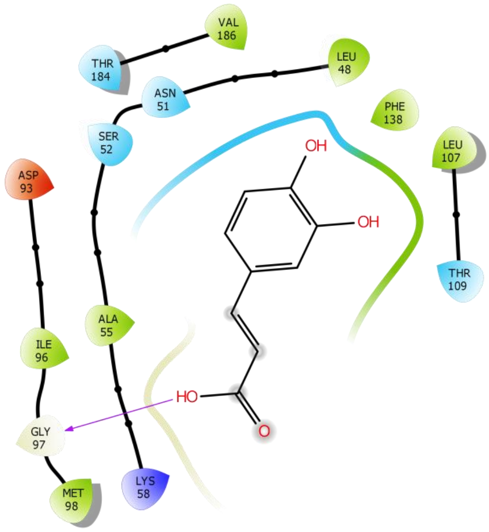

Caffeic acid 2D diagrams of docked conformation compound with 2VCZ

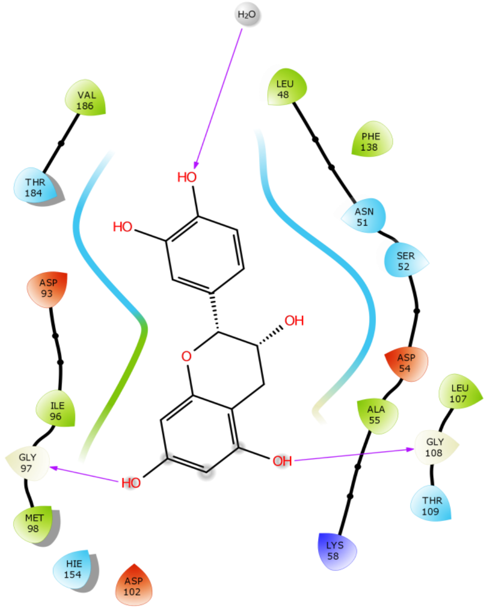

Catechin 2D diagrams of docked conformation compound 2VCZ

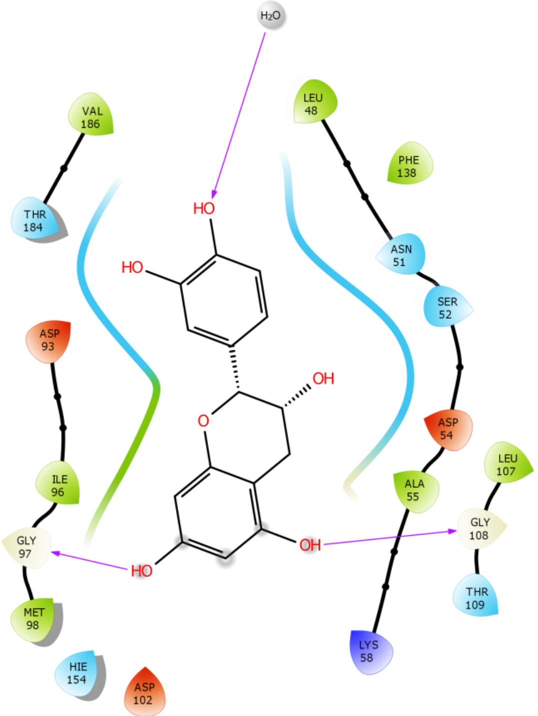

Epicatechin **2D diagrams of docked conformation compound with 2VCZ**

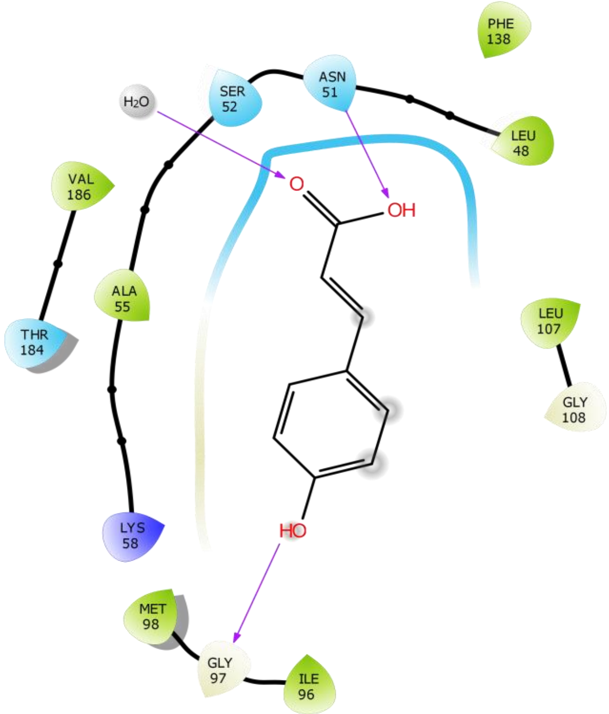

Trans coumaric acid 2D diagrams of docked conformation compound 2VCZ

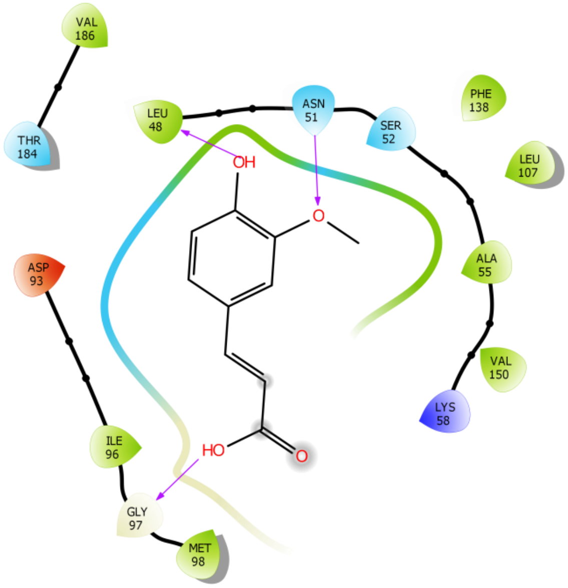

Trans ferullic acid **2D diagrams of docked conformation compound with 2VCZ**

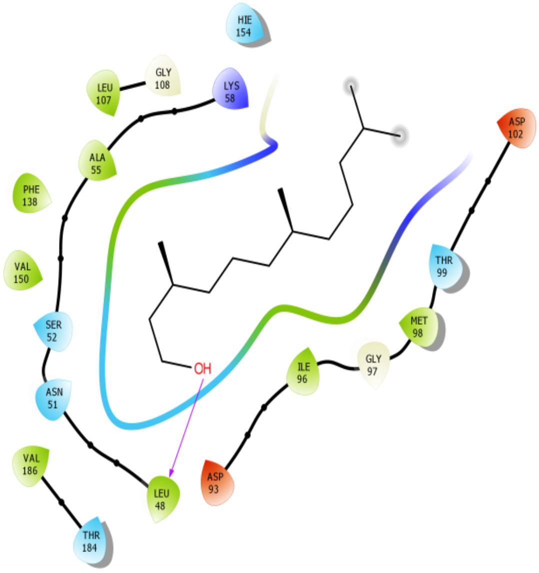

3,7,11-Trimethyl-1-dodecanol **2D diagrams of docked conformation compound 2VCZ**

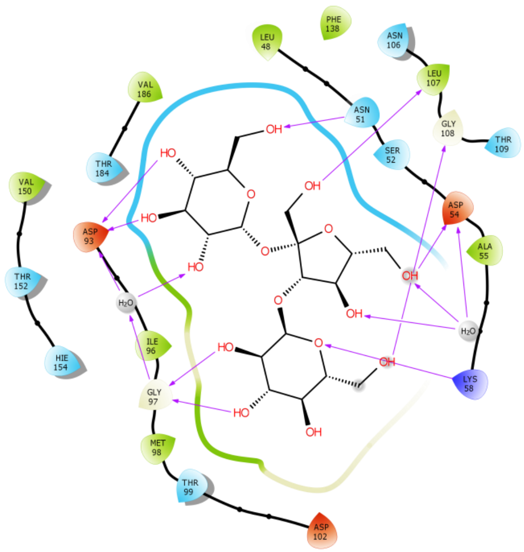

Melezitose 2D diagrams of docked conformation compound with 2VCZ

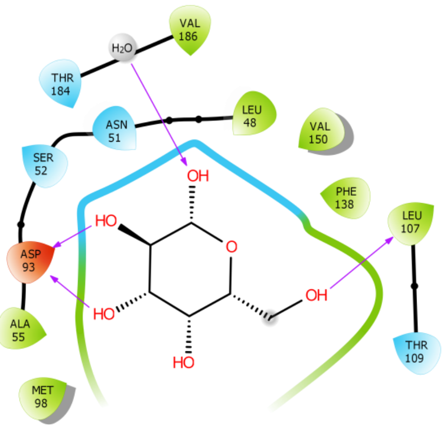

beta-lactose 2D diagrams of docked conformation compound 2VCZ

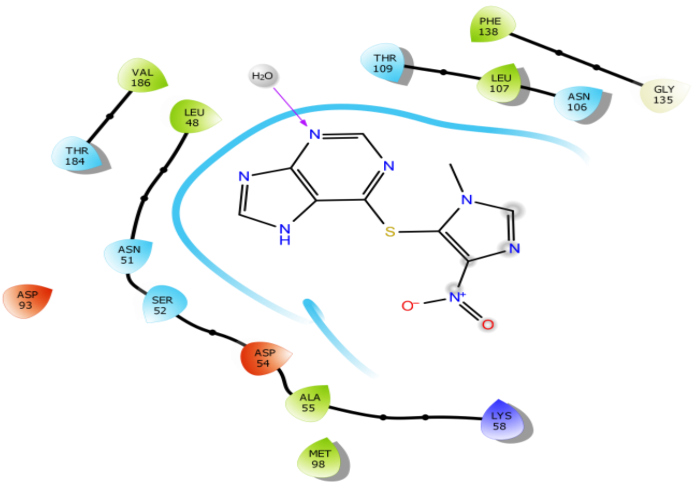

Aziothioprine 2D diagrams of docked conformation compound with 2VCZ

Figure S6:

PDB ID 1G1T: Crystal Structure of E-Selectin Lectin/Egf Domains Complexed with Slex

Classification: IMMUNE SYSTEM, MEMBRANE PROTEIN

Organism(s): Homo sapiens

Expression system: Cricetulus griseus

Mutation(s): No

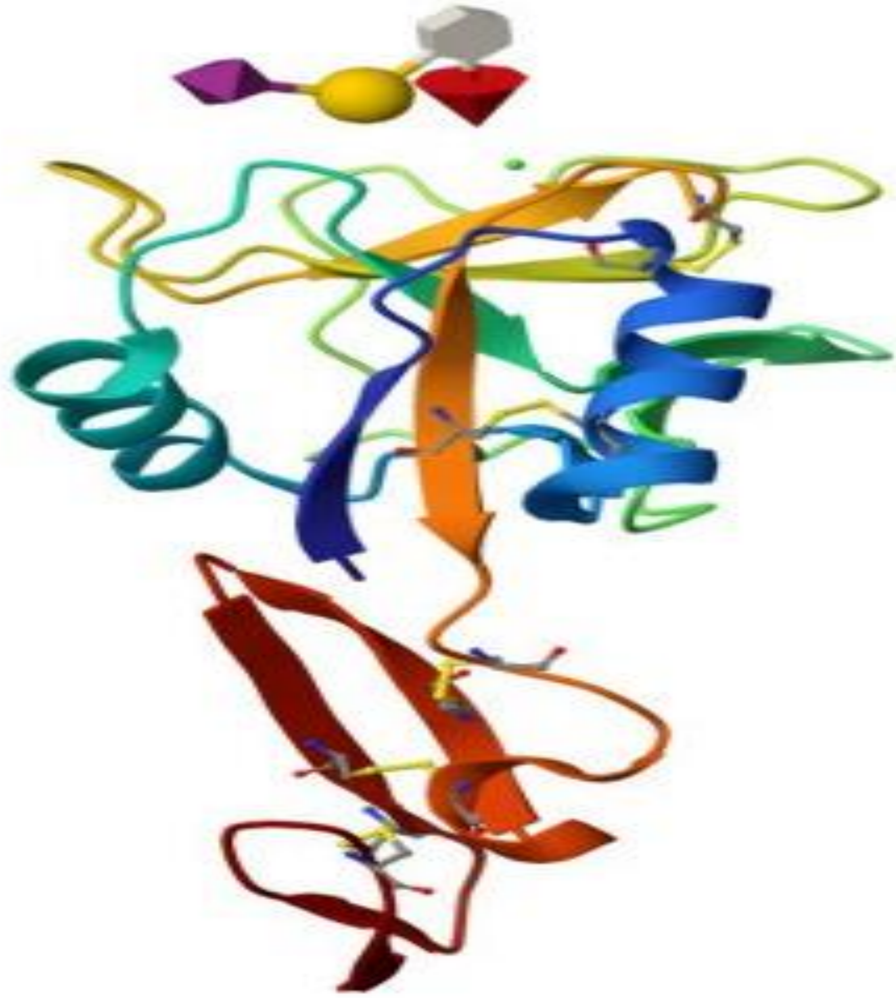

3D- Structure of protein (1G1T)

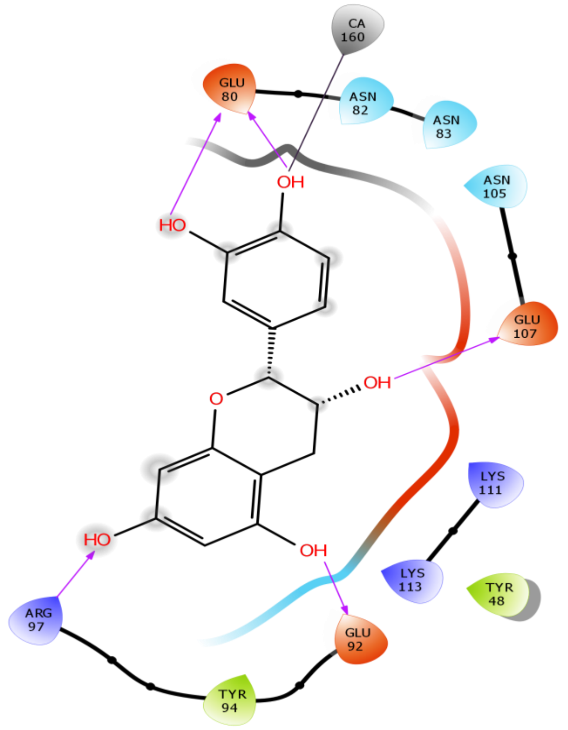

Catechin 2D diagrams of docked conformation compound with (1G1T)

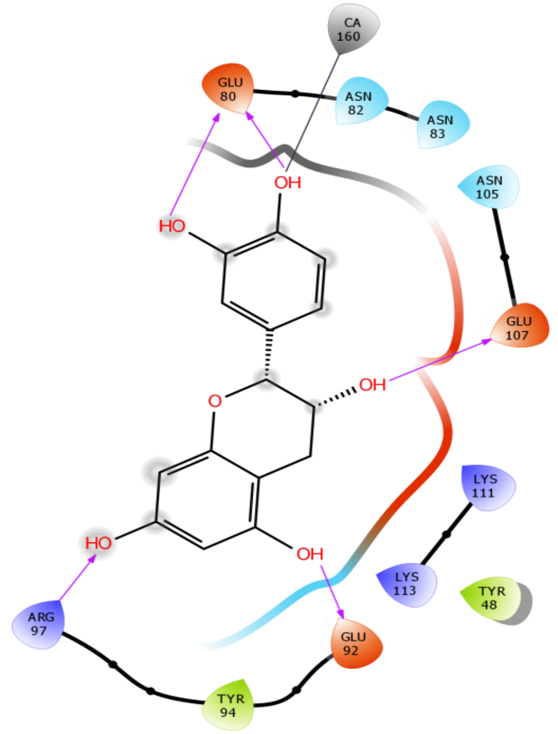

Epicatechin 2D diagrams of docked conformation compound with (1G1T)

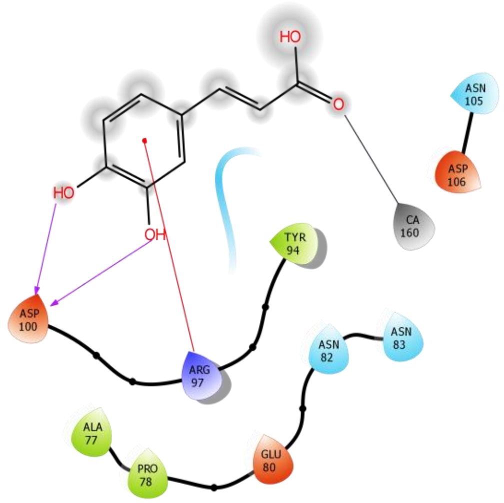

Caffeic acid 2D diagrams of docked conformation compound with (1G1T)

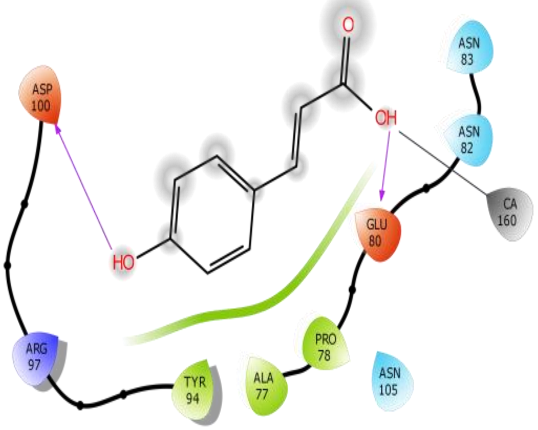

Trans coumaric acid 2D diagrams of docked conformation compound with (1G1T)

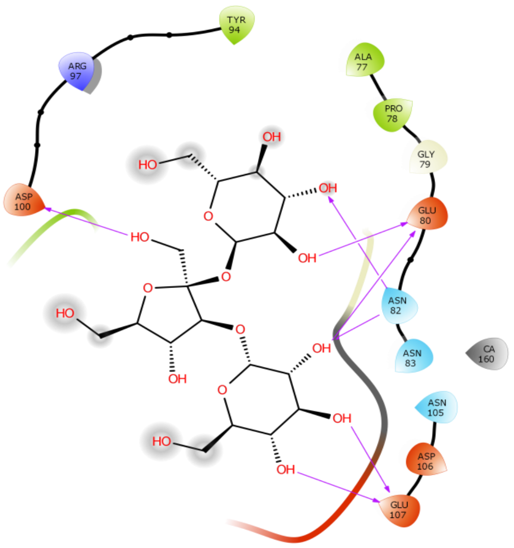

Melezitose 2D diagrams of docked conformation compound with (1G1T)

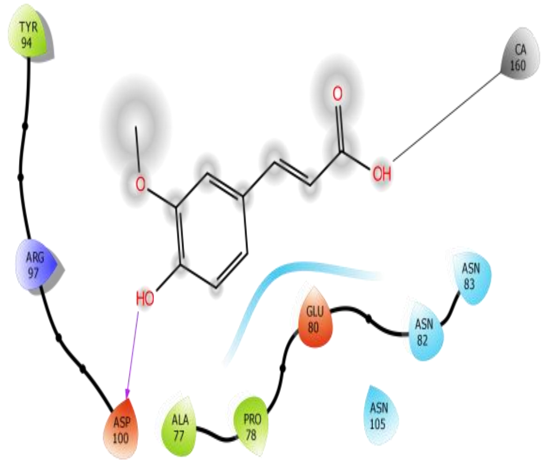

Trans coumaric acid 2D diagrams of docked conformation compound with (1G1T)

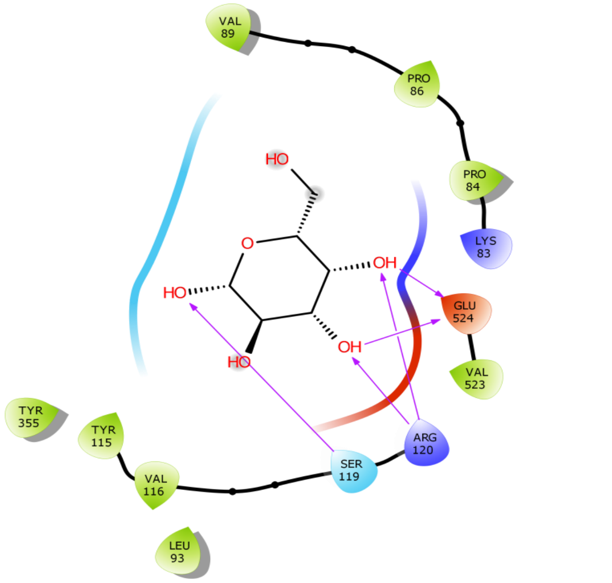

Beta-lactose 2D diagrams of docked conformation compound with (1G1T)

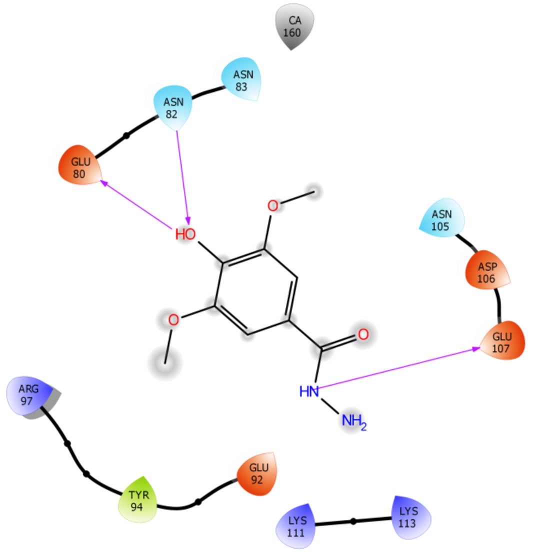

4-Hydroxy-3,5-dimethoxybenzohydrazide 2D diagrams of docked conformation compound with (1G1T)

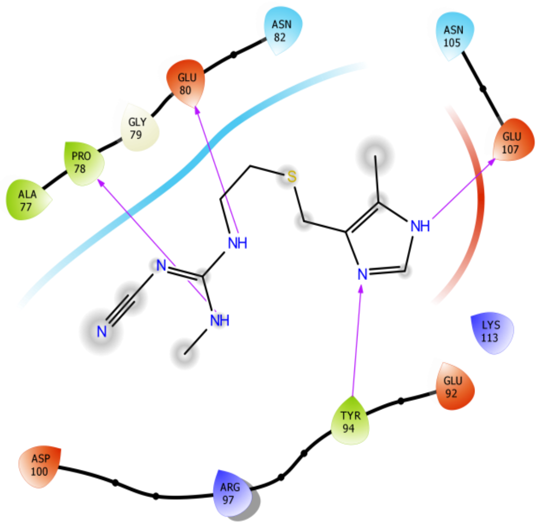

Cimetidine 2D diagrams of docked conformation compound with (1G1T)

Figure S7:

PDB ID 6BL4: Crystal Complex of Cyclooxygenase-2 with indomethacin-ethylenediamine-dansyl conjugate.

Classification: OXIDOREDUCTASE/INHIBITOR

Organism(s): Mus musculus

Expression system: Spodoptera frugiperda

Mutation(s): No

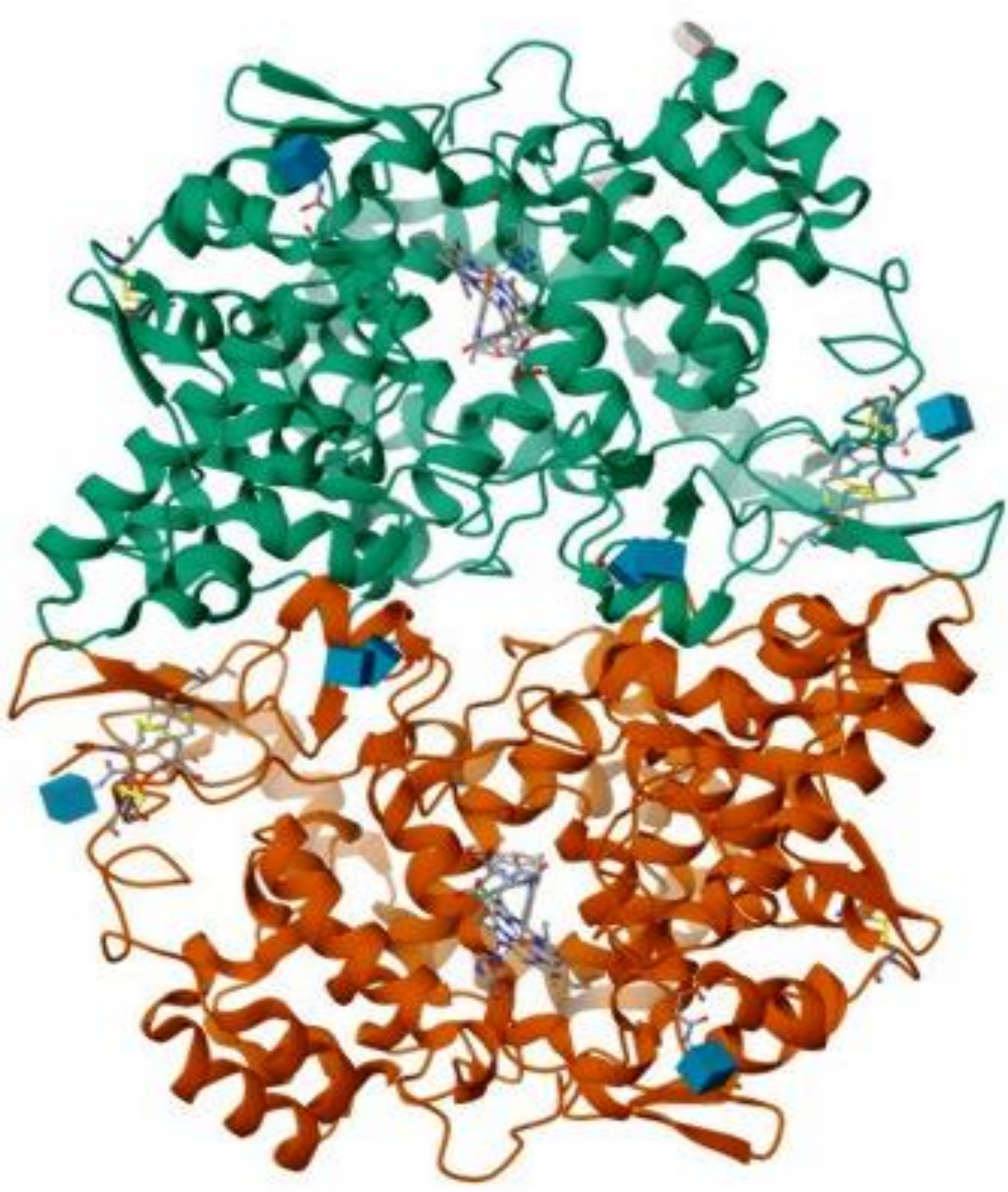

3D- Structure of protein (6BL4)

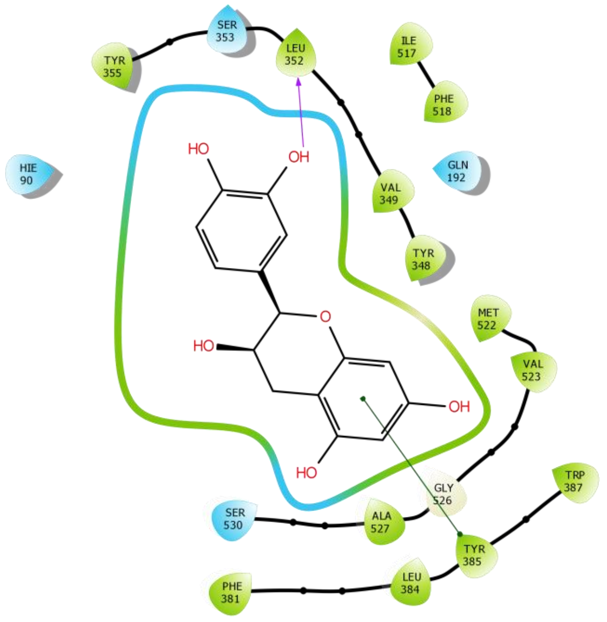

Catechin diagrams of docked conformation compound with 6BL4

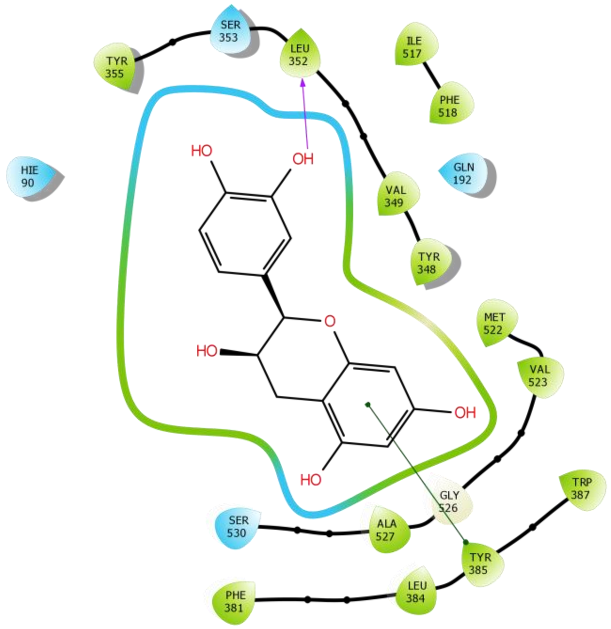

Epicatechin 2D diagrams of docked conformation compound with 6BL4

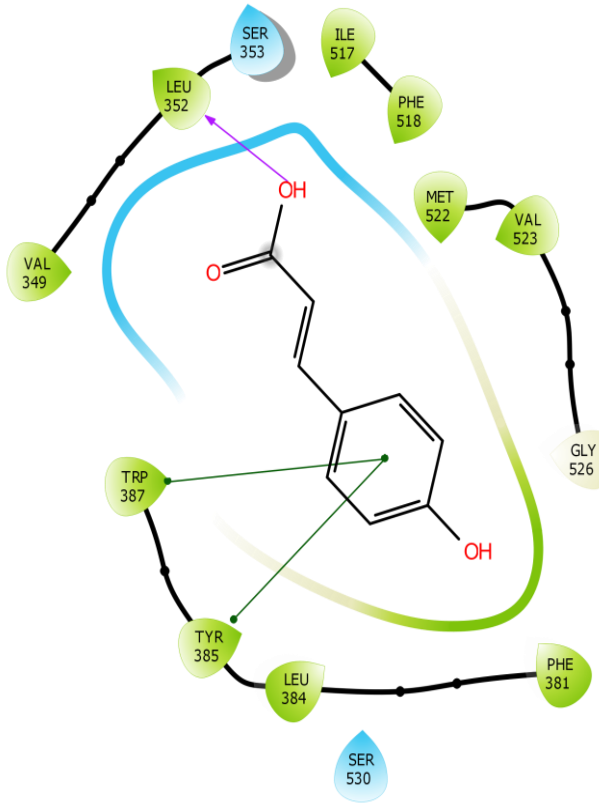

Trans-p-coumaric acid **diagrams of docked conformation compound with 6BL4**

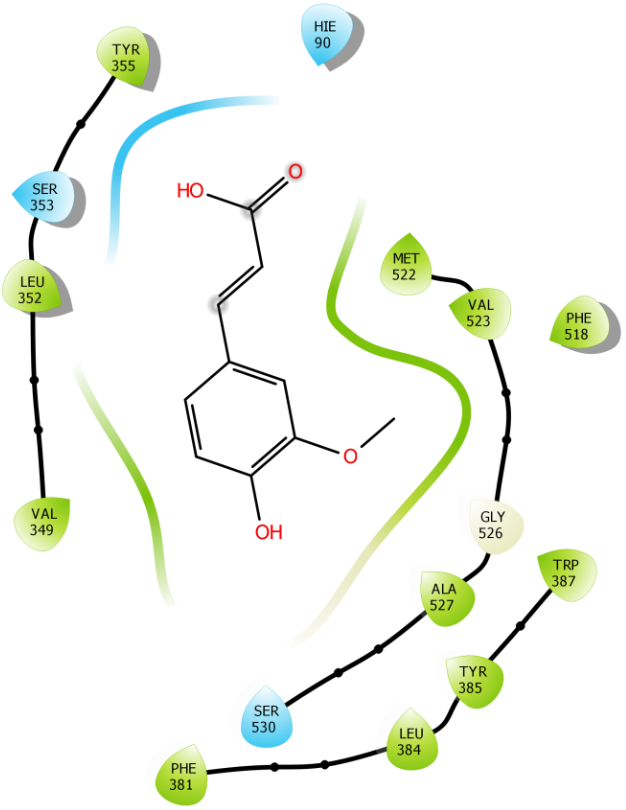

Trans-ferulic acid 2D diagrams of docked conformation compound with 6BL4

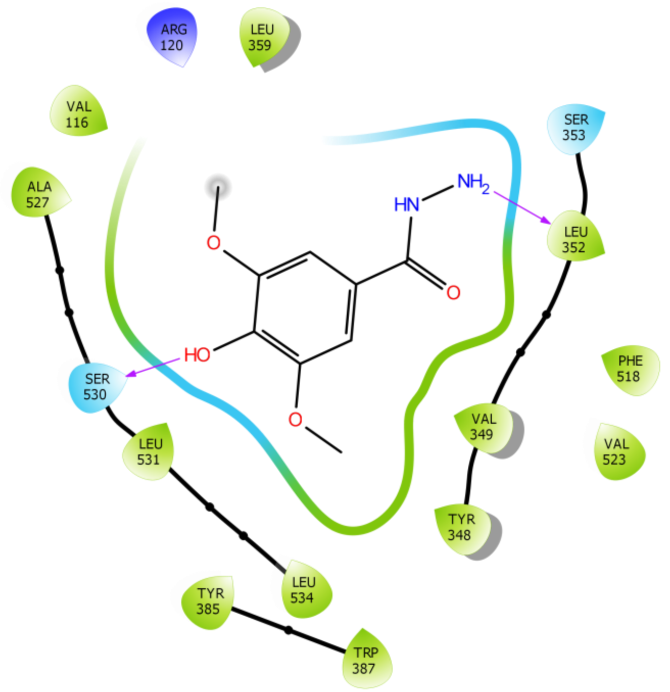

4-Hydroxy-3,5-dimethoxybenzohydrazide acid 2D diagrams of docked conformation compound with 6BL4

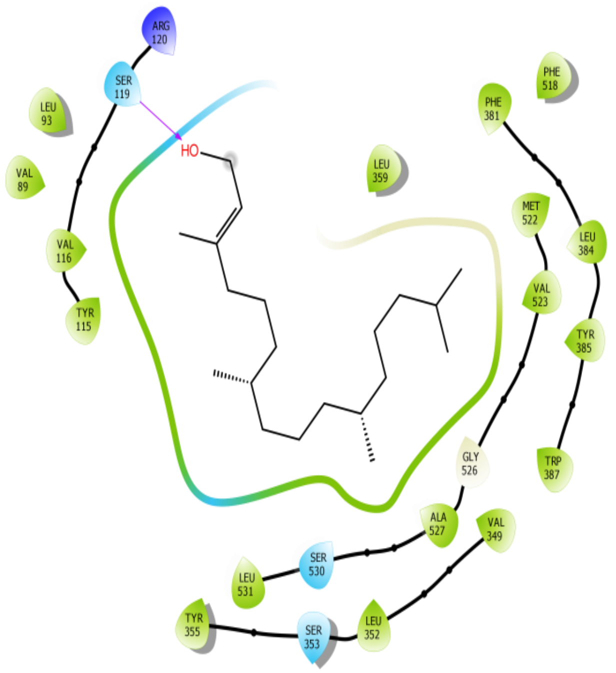

3,7,11,15-Tetramethyl-2-hexadecen-1-ol, 2D diagrams of docked conformation compound with 6BL4

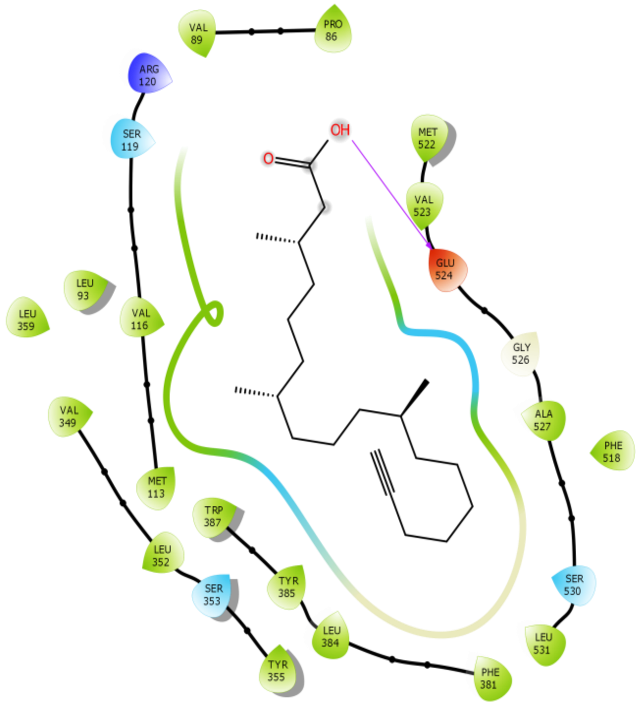

Fig. 138: 3,7,11-trimethyl-17-Octadecynoic acid 2D diagrams of docked conformation compound with 6BL4

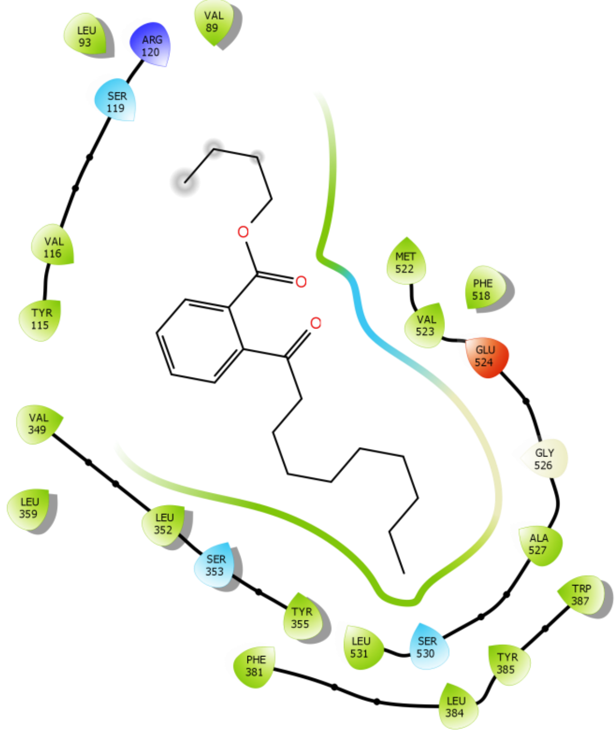

Butyl octyl phthalate 2D diagrams of docked conformation compound with 6BL4

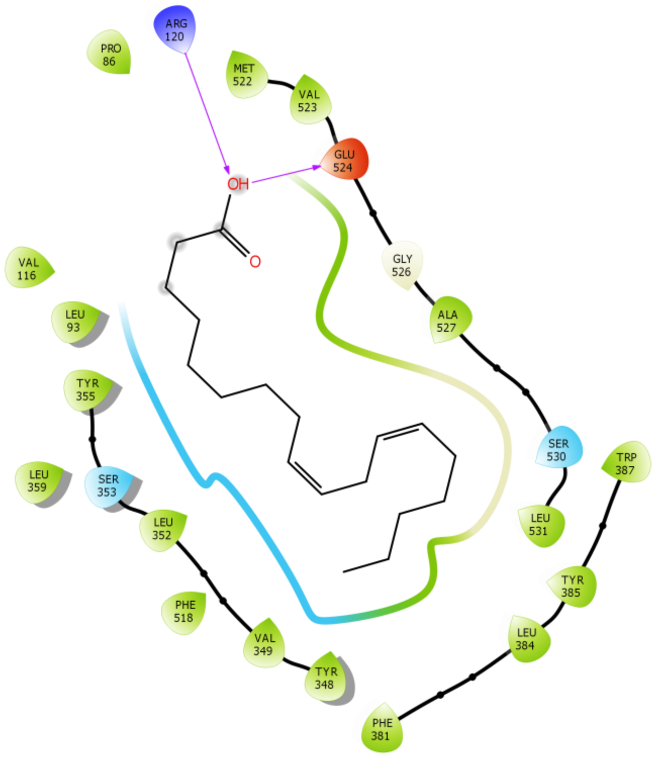

Linolenic acid 2D diagrams of docked conformation compound with 6BL4

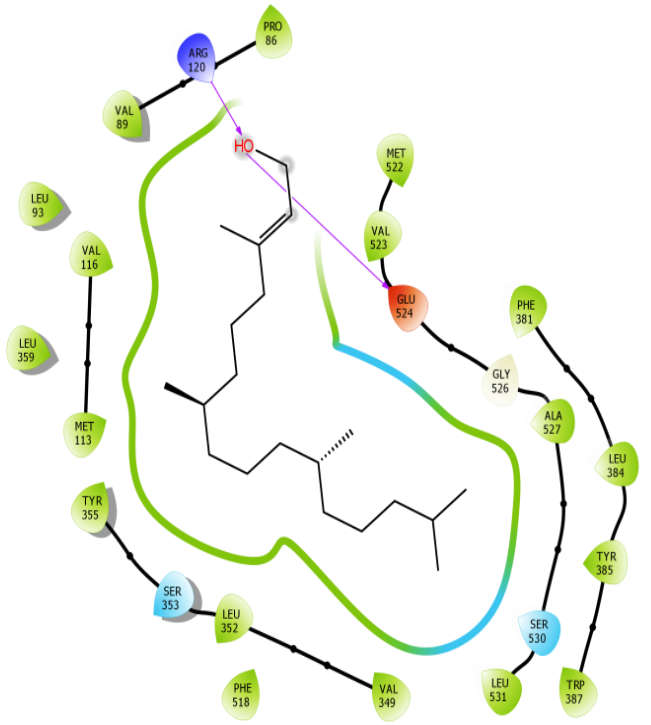

Phytol ether 2D diagrams of docked conformation compound with 6BL4

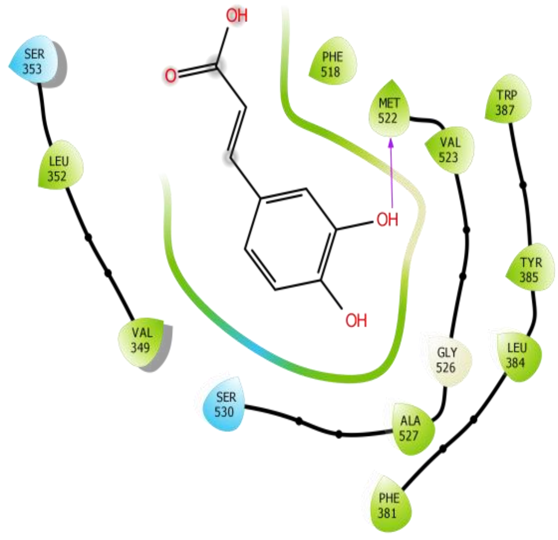

Caffeic acid 2D diagrams of docked conformation compound with 6BL4

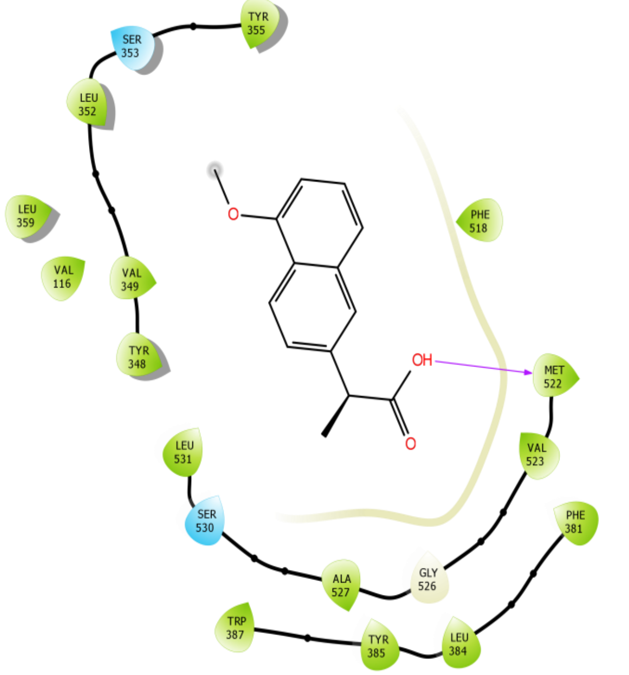

Aspirin 2D diagrams of docked conformation compound with 6BL4

Figure S8:

PDB ID 1OG5: Structure of human cytochrome P450 CYP2C9

Classification: ELECTRON TRANSPORT

Organism(s): Homo sapiens

Expression system: Escherichia coli

Mutation(s): YES

Membrane Protein: Yes

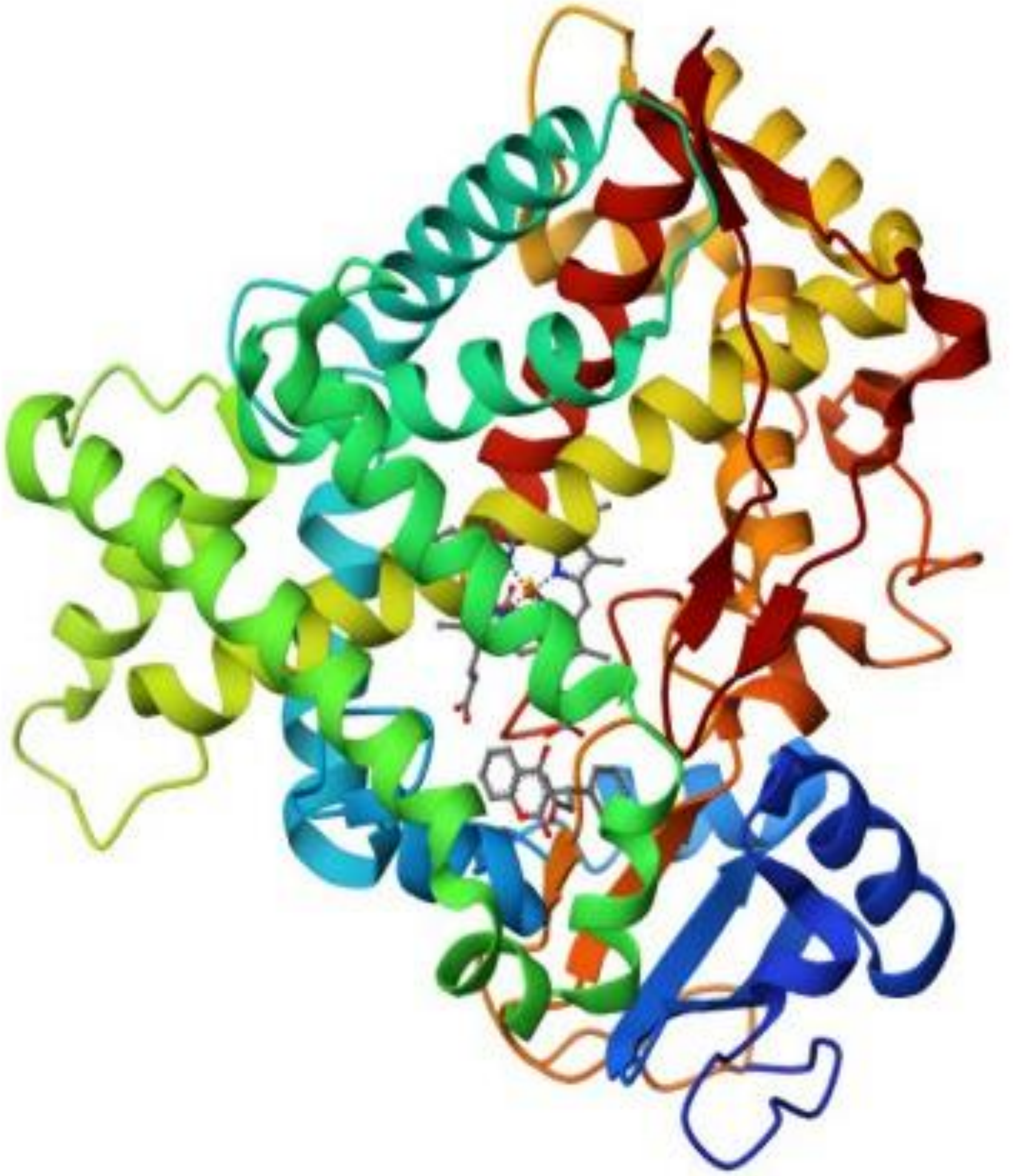

3D- Structure of protein (1OG5)

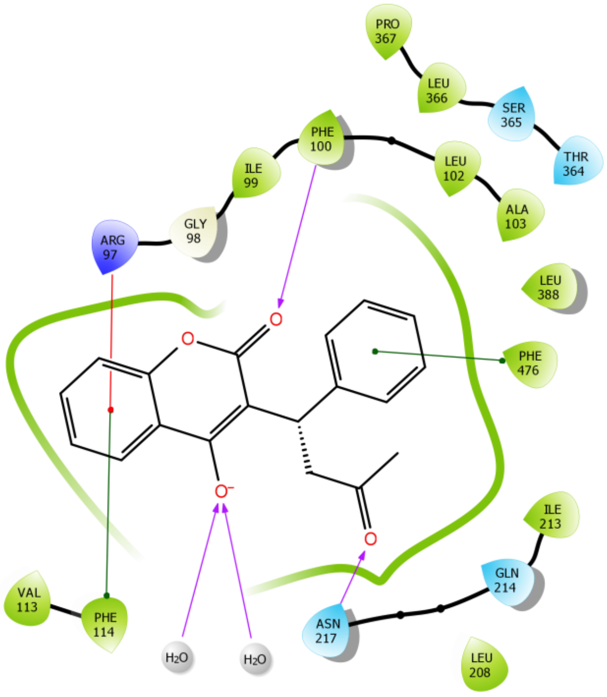

Trans-p-coumaric acid diagrams of docked conformation compound with 1OG5

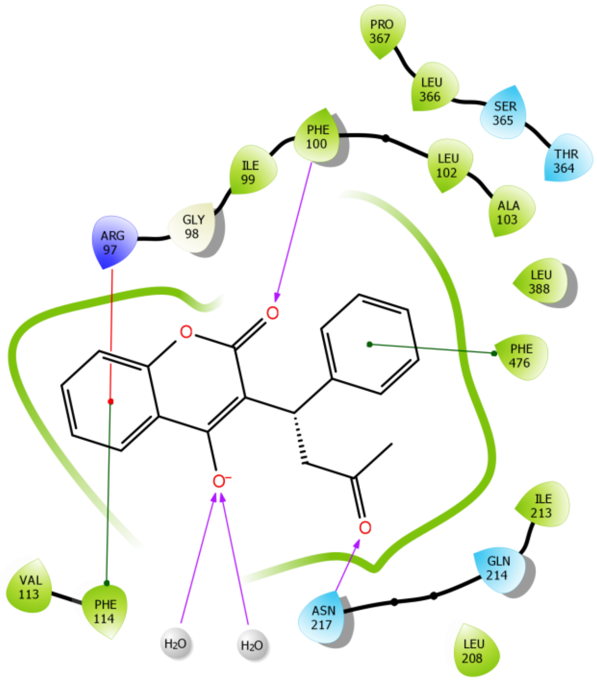

Trans-ferulic acid 2D diagrams of docked conformation compound with 1OG5

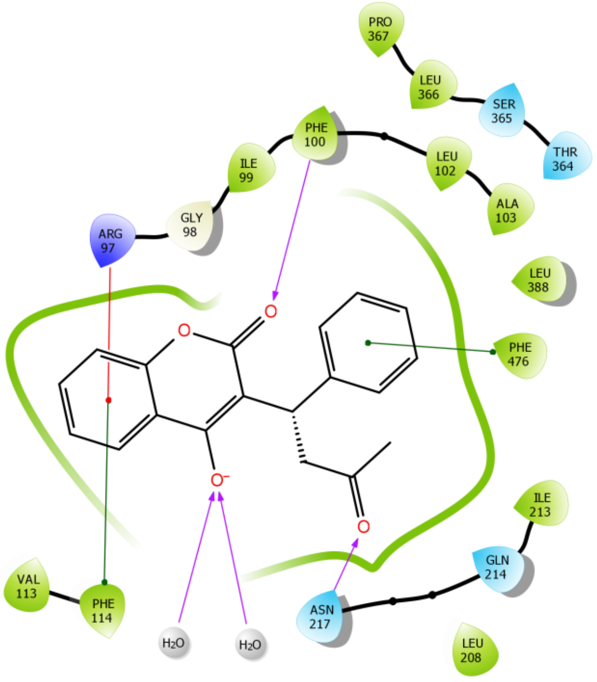

3,7,11-Trimethyl-1-dodecanol 2D diagrams of docked conformation compound with 1OG5

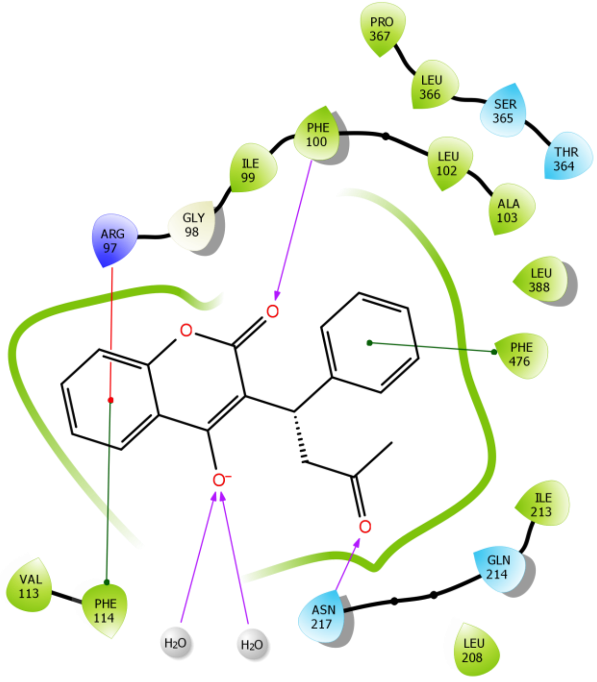

Melezitose 2D diagrams of docked conformation compound with 1OG5

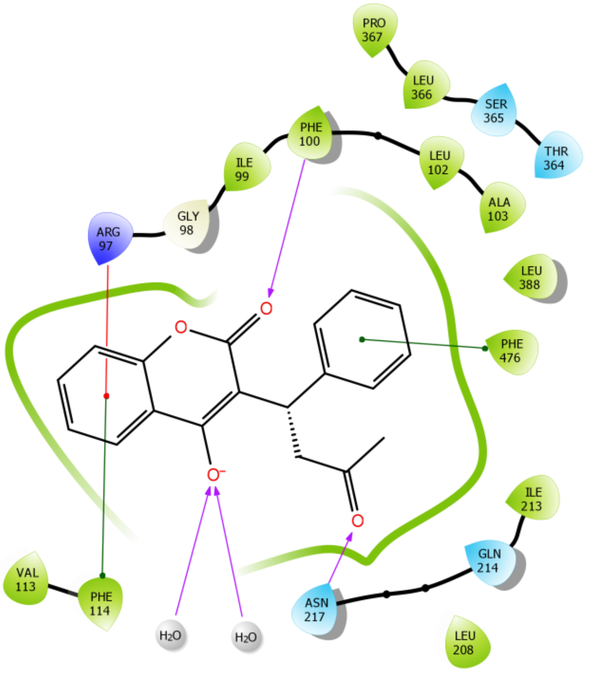

beta-hydroxydodecanoic acid 2D diagrams of docked conformation compound with 1OG5

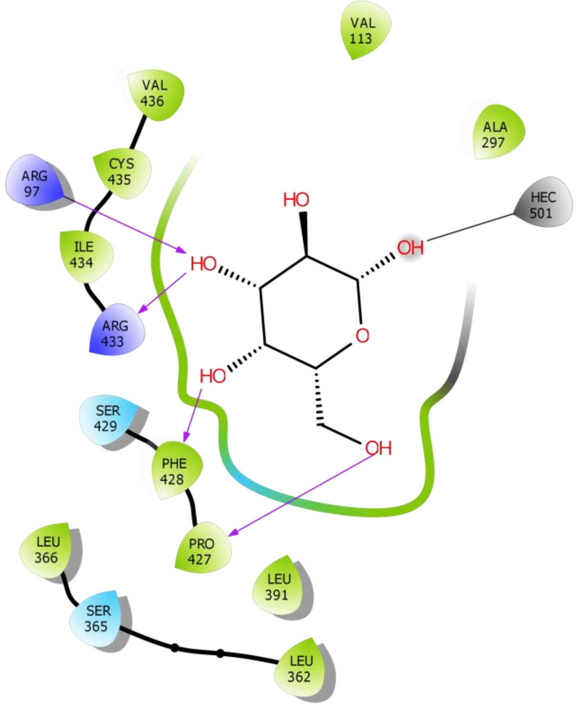

beta-lactose 2D diagrams of docked conformation compound with 1OG5

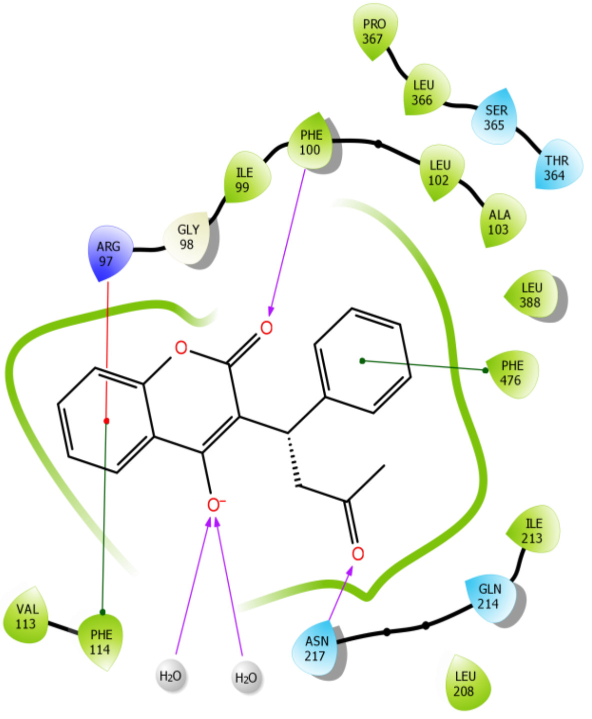

4-Hydroxy-3,5-dimethoxybenzohydrazide acid 2D diagrams of docked conformation compound with 1OG5

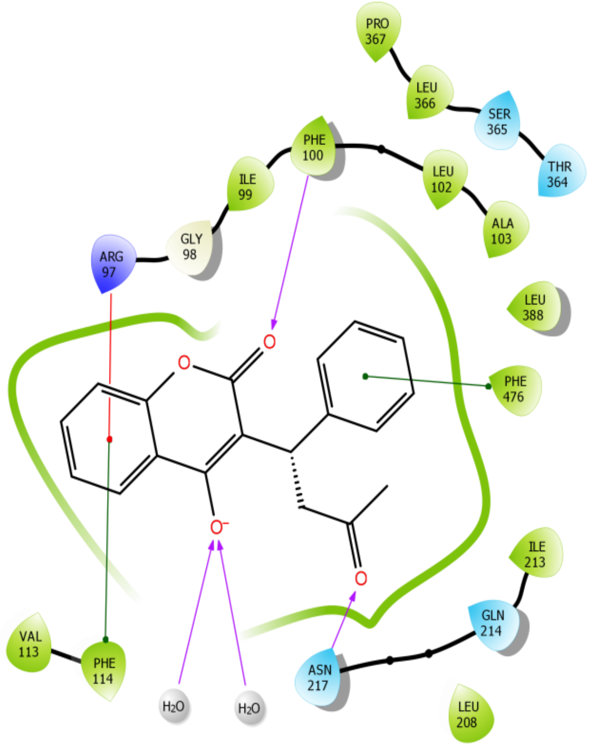

1-Dodecanol 2D diagrams of docked conformation compound with 1OG5

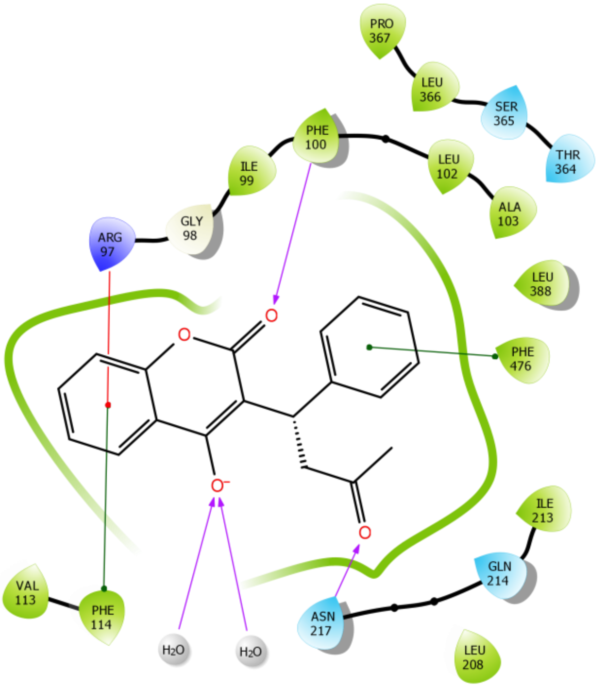

17-octadecynoic acid 2D diagrams of docked conformation compound with 1OG5

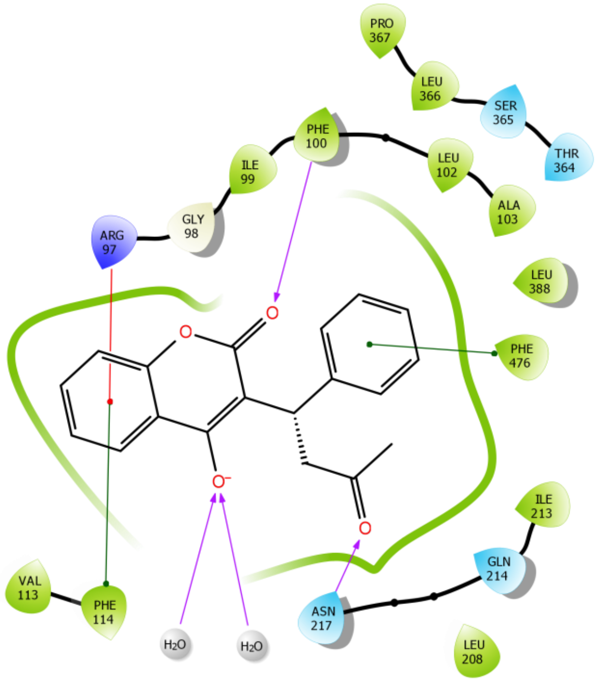

13-Heptadecyn-1-ol acid 2D diagrams of docked conformation compound with 1OG5

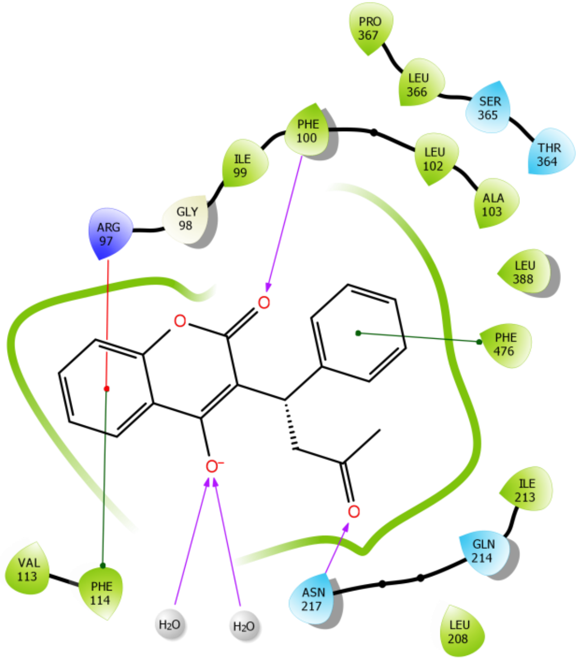

Palmitic acid 2D diagrams of docked conformation compound with 1OG5

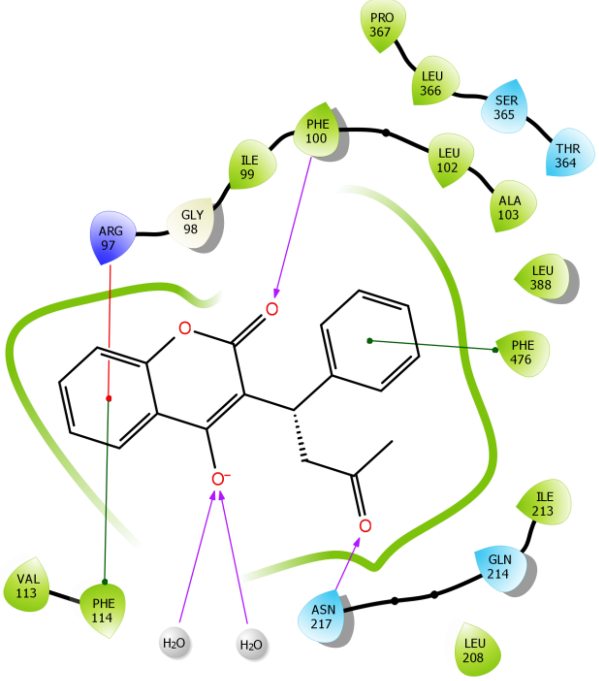

Butyl octyl phthalate 2D diagrams of docked conformation compound with 1OG5

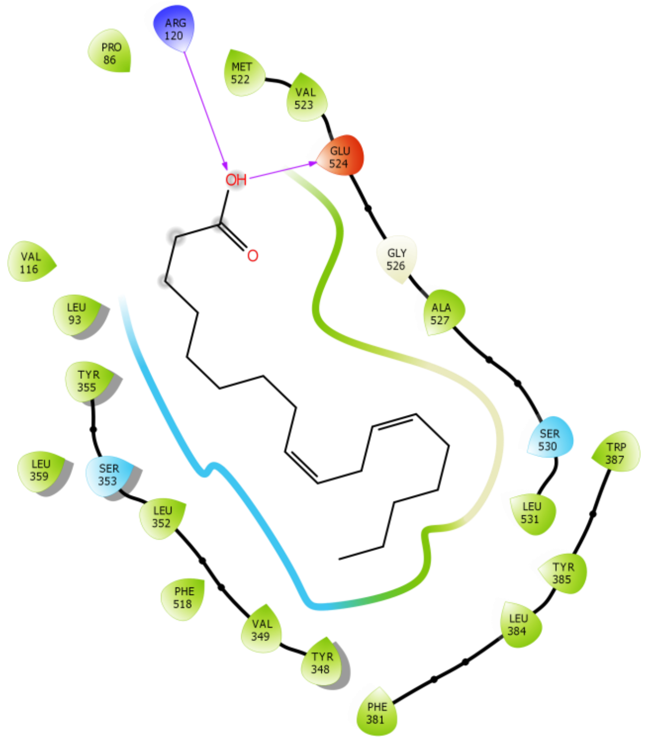

Linolenic acid 2D diagrams of docked conformation compound with 1OG5

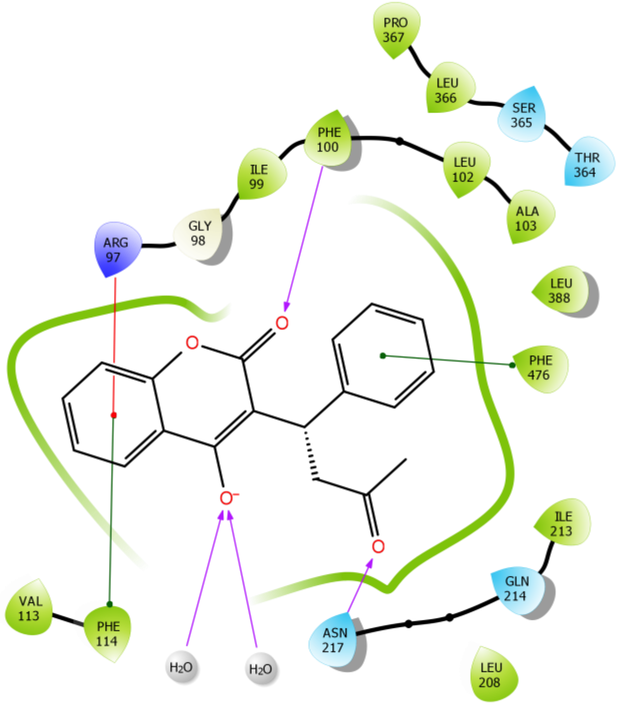

Stearyl vinyl ether 2D diagrams of docked conformation compound with 1OG5

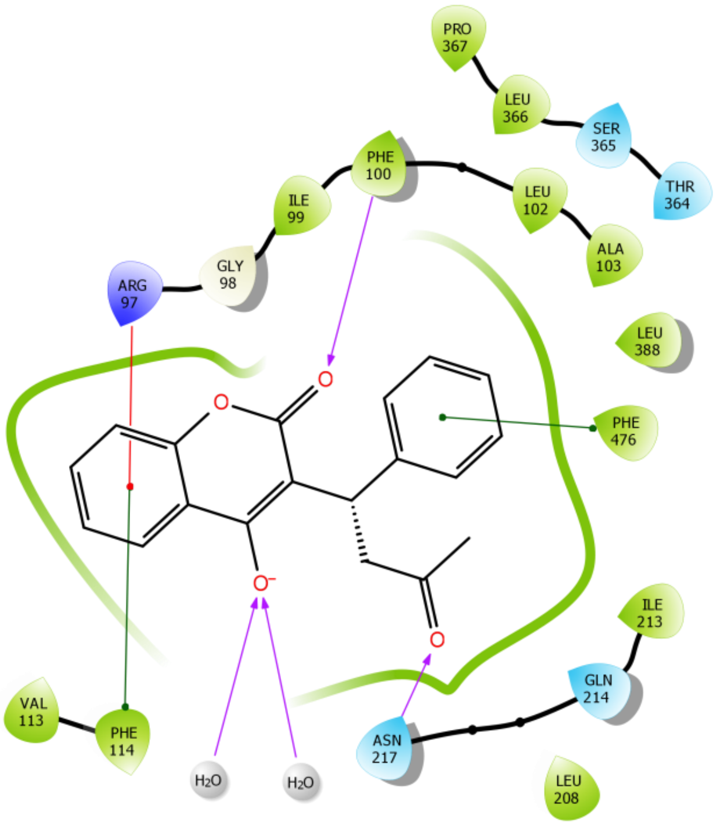

Trolox 2D diagrams of docked conformation compound with 1OG5

Figure S9:

PDB ID 5X14: Crystal structure of Bacillus subtilis PadR in complex with ferulic acid

Classification: TRANSCRIPTION

Organism(s): Bacillus subtilis subsp. spizizenii str. W23

Expression system: Escherichia coli

Mutation(s): No

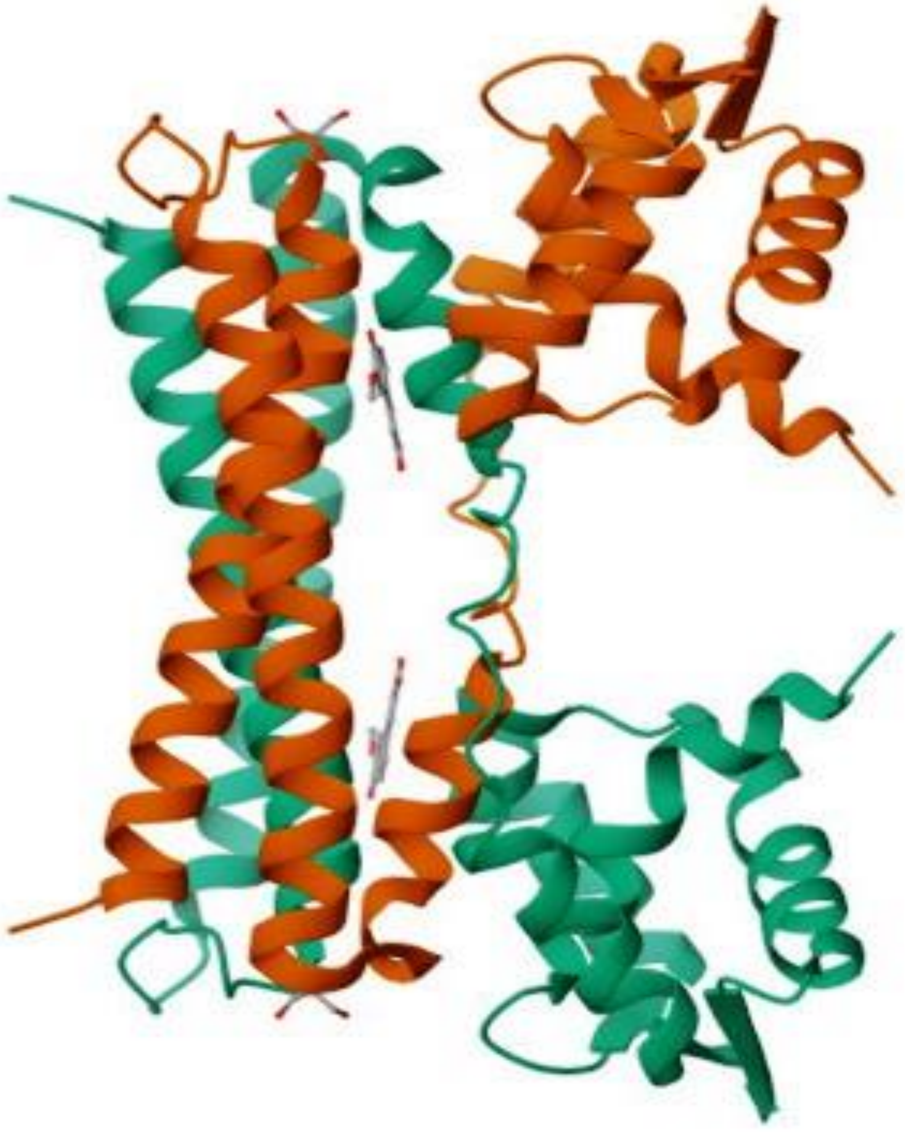

3D- Structure of protein (5X14)

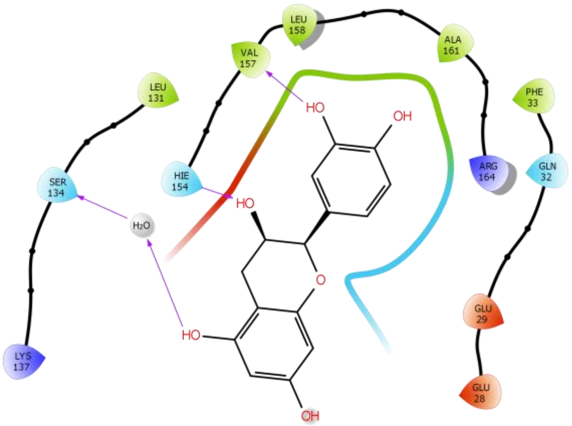

Catechin 2D diagrams of docked conformation compound with 5X14

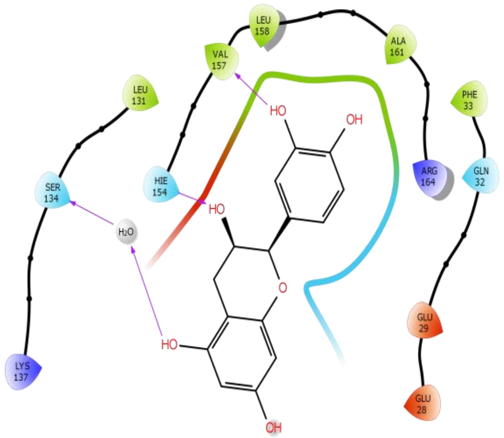

Epicatechin 2D diagrams of docked conformation compound with 5X14

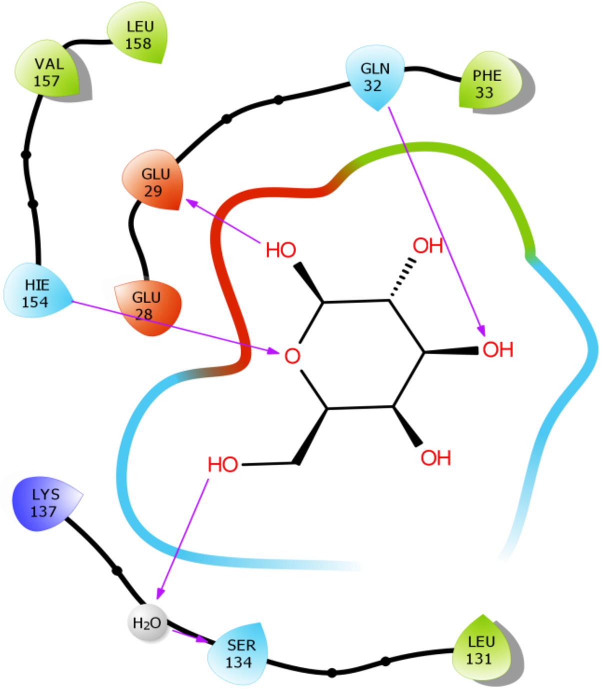

beta-lactose 2D diagrams of docked conformation compound with 5X14

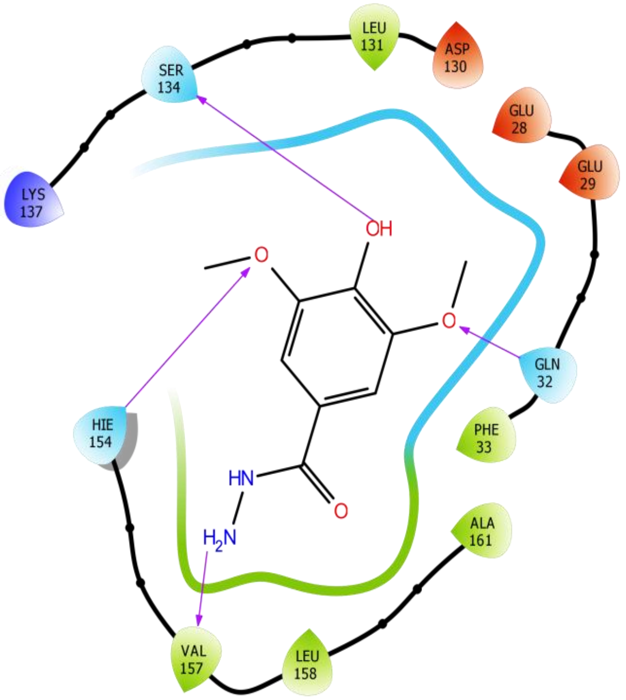

4-Hydroxy-3,5-dimethoxybenzohydrazide 2D diagrams of docked conformation compound with 5X14

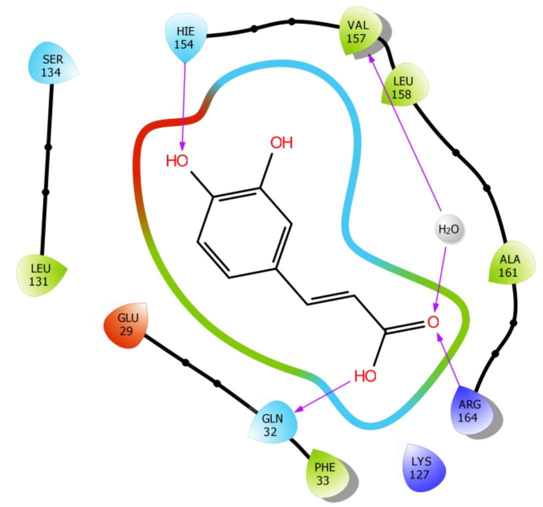

Caffeic acid 2D diagrams of docked conformation compound with 5X14

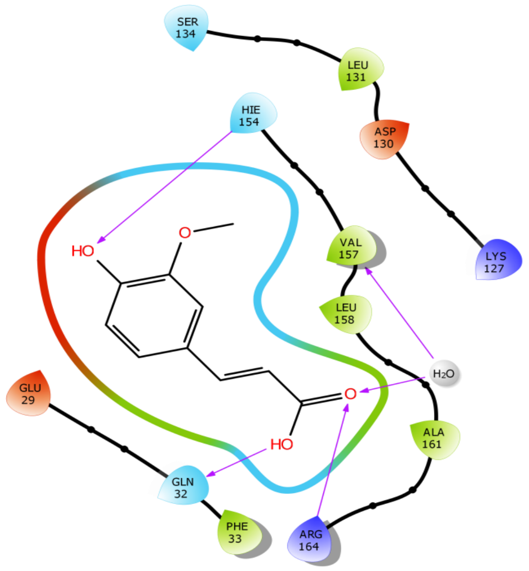

Trans ferulic acid 2D diagrams of docked conformation compound with 5X14

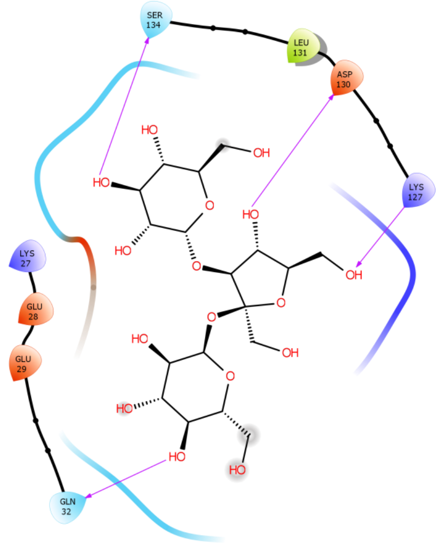

Melezitose 2D diagrams of docked conformation compound with 5X14

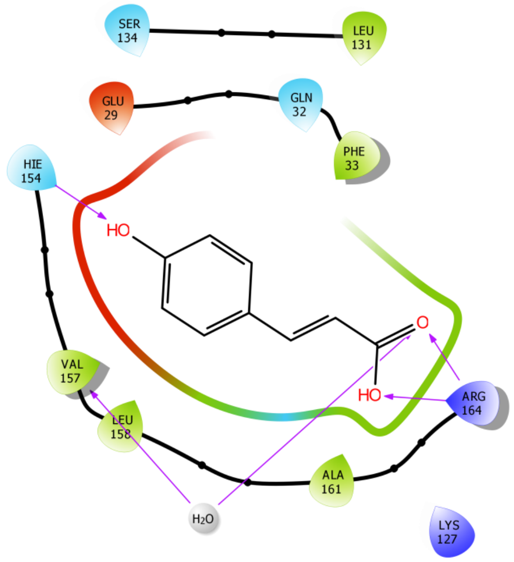

Trans-p-coumaric acid **2D diagrams of docked conformation compound with 5X14**

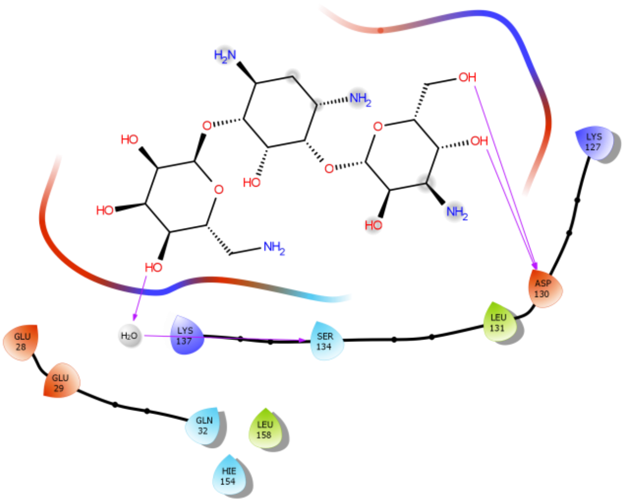

Kanamycin 2D diagrams of docked conformation compound with 5X14
